# Disease and Health Inequalities Attributable to Air Pollutant Exposure in Detroit, Michigan

**DOI:** 10.3390/ijerph14101243

**Published:** 2017-10-19

**Authors:** Sheena E. Martenies, Chad W. Milando, Guy O. Williams, Stuart A. Batterman

**Affiliations:** 1 Environmental Health Sciences, University of Michigan School of Public Health, 1415 Washington Heights, Ann Arbor, MI 48109, USA; smarten@umich.edu (S.E.M.); cmilando@umich.edu (C.W.M.); 2 Detroiters Working for Environmental Justice, 4750 Woodward Ave., Suite 415, Detroit, MI 48201, USA; guy@detroitenvironmentaljustice.org

**Keywords:** ambient air pollution, burden of disease, health impact assessment, urban health

## Abstract

The environmental burden of disease is the mortality and morbidity attributable to exposures of air pollution and other stressors. The inequality metrics used in cumulative impact and environmental justice studies can be incorporated into environmental burden studies to better understand the health disparities of ambient air pollutant exposures. This study examines the diseases and health disparities attributable to air pollutants for the Detroit urban area. We apportion this burden to various groups of emission sources and pollutants, and show how the burden is distributed among demographic and socioeconomic subgroups. The analysis uses spatially-resolved estimates of exposures, baseline health rates, age-stratified populations, and demographic characteristics that serve as proxies for increased vulnerability, e.g., race/ethnicity and income. Based on current levels, exposures to fine particulate matter (PM_2.5_), ozone (O_3_), sulfur dioxide (SO_2_), and nitrogen dioxide (NO_2_) are responsible for more than 10,000 disability-adjusted life years (DALYs) per year, causing an annual monetized health impact of $6.5 billion. This burden is mainly driven by PM_2.5_ and O_3_ exposures, which cause 660 premature deaths each year among the 945,000 individuals in the study area. NO_2_ exposures, largely from traffic, are important for respiratory outcomes among older adults and children with asthma, e.g., 46% of air-pollution related asthma hospitalizations are due to NO_2_ exposures. Based on quantitative inequality metrics, the greatest inequality of health burdens results from industrial and traffic emissions. These metrics also show disproportionate burdens among Hispanic/Latino populations due to industrial emissions, and among low income populations due to traffic emissions. Attributable health burdens are a function of exposures, susceptibility and vulnerability (e.g., baseline incidence rates), and population density. Because of these dependencies, inequality metrics should be calculated using the attributable health burden when feasible to avoid potentially underestimating inequality. Quantitative health impact and inequality analyses can inform health and environmental justice evaluations, providing important information to decision makers for prioritizing strategies to address exposures at the local level.

## 1. Introduction

### 1.1. Background

Cumulative impact analyses aim to understand the way social and environmental factors combine to increase adverse health risks and impacts across a population [1]. This information can identify areas where social and environmental stressors together create environmental justice (EJ) concerns, such as disproportionate impacts and health disparities among low income communities and communities of color [2], often with the goal of helping disadvantaged groups gain access to the resources needed to improve existing conditions [1]. These studies often focus on susceptible and vulnerable populations. Susceptibility typically refers to intrinsic factors that tend to intensify the biological response that results from exposure to a stressor, such as advanced age or underlying disease; vulnerability typically refers to extrinsic factors that can increase exposures or reduce the ability to mitigate them, such as living near a pollutant source or having lower socioeconomic status (SES) [3,4]. Disproportionate impacts can result where exposures are high and residents are susceptible or vulnerable.

Cumulative impact analysis frameworks, which are intended to quantify the degree to which segments of the population are disproportionately impacted [5], have been developed to incorporate several social and environmental hazards, e.g., air pollutants, temperature, high rates of disease, and proximity to hazardous land uses. These studies often use a weighted index or similar metric to combine factors into a single score that can be used to compare burdens across groups. Air pollution is a frequently cited environmental hazard in cumulative impact assessments, e.g., disproportionate impacts from exposures to nitrogen oxides (NO_x_), fine particulate matter (PM_2.5_), and diesel particulate matter (DPM) have been shown at the census tract level for minority populations in California [6,7], and for traffic-related exposures among non-white and low SES populations in Minneapolis [8]. Using exposures as a proxy for air pollution health impacts, however, may be problematic for several reasons. First, many cumulative impact studies use poorly-resolved exposure data. For example, estimating exposures using distance-weighted concentrations at the nearest ambient monitoring station [9] may poorly represent intra-urban gradients in exposure that affect the distribution of impacts [10,11]. Second, exposures alone do not account for vulnerability factors that can increase the risk of an adverse health impact [5]. These factors are especially important for pollutants that have limited spatial variability, e.g., ozone (O_3_); for these pollutants, inequalities will be driven by differences in susceptibility or vulnerability rather than exposure. Other issues with using exposures as a proxy for health risks include the difficulty in assigning weights to pollutants that have different health effects [12], the limited ability to assess exposures to multiple pollutants, and difficulty of identifying culpable sources or source categories.

There is a growing effort to incorporate cumulative impact analyses into regulatory and decision-making processes to advance policy goals and public health initiatives [13]. One approach is to expand the use of quantitative health impact assessment (HIA) methods to better include equity concerns. Quantitative HIAs combine information on population exposures, baseline health rates, concentration-response functions, and other data to estimate the fraction of health impacts attributable to exposures. HIAs are becoming preferred tools for decision making, and several applications have included ambient air pollution as an important environmental exposure [14]. HIA techniques are routinely used to help set the National Ambient Air Quality Standard (NAAQS) [15,16,17,18]. HIAs for air pollution have estimated the health burden in the USA attributable to PM_2.5_ and O_3_ exposures, which totals 130,000 premature deaths, 180,000 hospitalizations and emergency department (ED) visits, and 100 million restricted activity days in the USA annually [19]. HIAs at the local scale, which incorporate more highly spatially-resolved exposure estimates and data on population susceptibility and vulnerability, have shown that health impacts are not evenly distributed and that socially disadvantaged populations often carry heavier burdens [20,21]. HIAs incorporating spatially explicit analyses of susceptibility and vulnerability factors can identify where pollutants have the greatest impact and which groups are most adversely affected. These analyses could support public health actions aimed at minimizing health burdens attributable to environmental exposures, representing a major transition from current practices that tend to be narrowly focused on compliance with regulations and standards such as the NAAQS.

### 1.2. Objectives

This study examines the health burden and health disparities (or inequities) attributable to air pollutant exposures at the urban scale. Impacts due to five pollutants (PM_2.5_, NO_2_, SO_2_, O_3_, and diesel exhaust particulate matter) are evaluated using HIA techniques and inequality metrics in a spatially-resolved analysis of Detroit, Michigan and neighboring cities. The analysis distinguishes impacts due to different types of sources, e.g., point (i.e., industrial) and mobile (i.e., on-road traffic) emission sources, and examines the sensitivity of results to spatial resolution and study boundaries.

Detroit and the surrounding communities make a compelling study location due to the density of heavy industry, historically high pollutant levels, and individual and population-level characteristics that increase vulnerability and susceptibility. A portion of the study area has been designated as non-attainment for the SO_2_ NAAQS, and the entire area is likely to be designated as non-attainment for O_3_ [22,23]. Area residents have high rates of diseases associated with environmental exposures, e.g., asthma hospitalization rates in the study area are nearly three times the state average [24], and characteristics that increase their vulnerability to air pollutants include proximity to industry, lower educational attainment, high rates of poverty, and linguistic isolation [25]. The study approach and many results are applicable to other EJ and cumulative impact analyses as well as environmental policy-making.

## 2. Materials and Methods

The health burden and disparities analyses use exposure information derived from air quality monitoring and dispersion modeling, quantitative HIA techniques, and inequality metrics, elements of which are described below. Additional details are in Appendix A.

### 2.1. Study Area, Spatial Resolution, and Study Population

**Figure 1:**
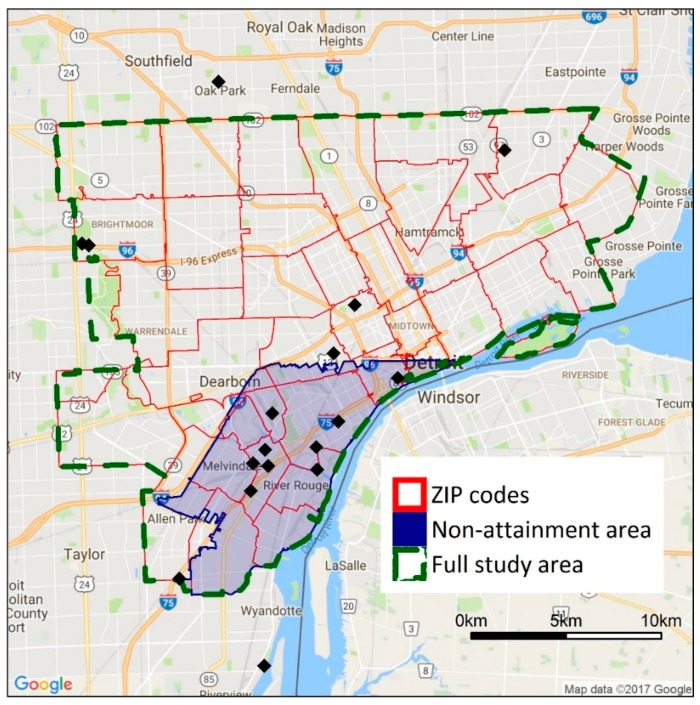
Map showing the full study area boundary and the study boundaries used in the sensitivity analyses. Black diamonds show the location of ambient air quality monitors in the area. The shaded area has been classified as non-attainment with the SO_2_ national ambient air quality standard (NAAQS).

The study area encompasses Detroit and the adjacent cities of Hamtramck, Highland Park, River Rouge, Ecorse, Lincoln Park, Melvindale, Dearborn, and Allen Park (Figure 1). This area has a total population of 945,000. Across the entire study area, 66% of residents identify as Black or African American, 7.3% identify as Hispanic or Latino, and 37% live below the poverty level [26]. In Detroit, which is the largest city in the study area, more than 92% of the population is non-white: 82.7% identify as Black or African American and 7.8% identify as Hispanic or Latino [27]. Across the study area, the percentage of persons in poverty ranges from 7.2% in Allen Park to 48.6% in Highland Park [26]. The percentage of the population that are persons of color is higher in the study area (75.6%) than in Wayne County (50.2%) and the state of Michigan as a whole (23.9%); similarly, the percentage of the population that lives below the poverty level in the study area (36.8%) is higher than in Wayne County (24.8%) and the state as a whole (23.7%) [26].

Selection of the study boundaries is based on several considerations. First, we focus on cities in southeast Michigan that may have higher exposures as a result of close proximity to industrial facilities and major highways or higher degrees of vulnerability and susceptibility, e.g., higher percentages of minority populations or populations in poverty; these cities have potentially high health burdens due to air pollutant exposures. Second, we use the municipal boundaries of each city to reflect the domain within which local decision makers may act. Third, the dispersion models discussed later are computationally intensive, and modeling larger study areas at a fine spatial resolution is impractical. Thus, a more focused analysis on the most heavily impacted cities in the Detroit metropolitan area is appropriate for this application. Limitations of this approach, especially for the inequality metrics, are examined in the Discussion Section.

Census blocks are selected as the unit of analysis for the exposure, health, and inequality metrics given the need to balance fine-scale exposure gradients with the availability of population and baseline health data, which are typically available only at coarser resolution, e.g., ZIP codes [28]. Exposures are based on residential location, following epidemiological studies from which the HIA concentration-response coefficients (discussed below) are drawn. Census block-level population data are taken from the 2010 census TIGER/Line shapefiles [29]. Block-level age-specific subgroups are estimated using the age distribution of the census block group based on the most recent five-year estimates (2010–2014) of the 2014 American Community Survey (ACS) [26].

### 2.2. Health Impact Assessment

The numbers of mortality and morbidity cases attributable to air pollution exposures are estimated using health impact functions which use baseline incidence rates, census block-level air pollutant concentrations, and concentration-response (CR) coefficients [30]. Conclusions of the most recent Integrated Science Assessments (ISA) [31,32,33,34,35] are used to select only those outcomes with established causal links. Exposure thresholds are not used because reliable population-level thresholds have not been identified in the epidemiologic literature [36,37,38]. The excess cancer risk attributable to diesel particulate matter (DPM) is estimated using methods described by Propper et al. [39]. Annual and daily concentrations are used in health impact functions, following the exposure estimates used in the original epidemiology studies, and annual concentrations are used in the estimates of excess cancer risk. Additional details on outcomes, concentration-response coefficients, and baseline outcome rates are presented in the Supplementary Materials.

The health burden is quantified using three metrics: the number of incident cases of mortality or morbidity attributable to pollutant exposure (attributable cases); disability-adjusted life years (DALYs); and monetized impacts. DALYs and monetized impacts are derived from the number of attributable cases. DALY calculations require a disability-weight (DW) and duration for each outcome [40]. Health impacts are monetized using valuations in the most recent PM_2.5_ standard analysis [16] and reported in 2010 dollars projected to a 2020 income level. DW, duration, and monetized values are found in the Appendix A.

### 2.3. Exposure Assessment

Spatially-resolved and current exposures of PM_2.5_, O_3_, NO_2_, and SO_2_ are estimated using air quality monitoring and dispersion modeling. Contributions from regional, point, mobile, and area sources are broken out separately.

Ambient air quality monitoring data from the USA and Canada for 2011–2015 were retrieved from USA and Canadian monitoring networks [41,42]. For PM_2.5_, we use 24 h averages from 12 sites in the Detroit area; two Canadian sites are excluded due to differences in measurement methods. For O_3_, we use hourly data from six sites in Detroit and two sites in Canada. Five of the six USA sites collected data only during the April to September period. Missing cold season hourly data at the five warm-season monitors are derived from data collected at the Allen Park site (USA) and the two Canadian sites using multiple imputation with predictive mean matching in R [43]. Hourly NO_2_ data are taken from five sites in Detroit and two in Canada, including two near-road sites. For SO_2_, hourly data from two monitoring sites in the USA and two in Canada operated throughout the study period are used; four additional sites around the Marathon Refinery collected data from 2014 onwards. Data from the year 2012 are used in the exposure assessment to coincide with the point and mobile source emissions inventories (discussed below).

Air quality dispersion modeling complements the exposures information provided by the monitoring data. Point source emissions of PM_2.5_, SO_2_, and NO_x_ are taken from the Michigan Air Emissions Reporting System [44] and the National Emissions Inventory [45]. The five-year average emission rate is used except for a few facilities that experienced large and known changes; these cases used the most recent years. Block-level concentrations of PM_2.5_, SO_2_, and NO_x_ from point sources are estimated using the software package Framework for Rapid Emissions Scenario and Health impact Estimation (FRESH-EST) [46], which uses a pre-computed source-receptor transfer coefficient matrix from the AERMOD dispersion model [47], local meteorology, and an adaptive receptor grid (200 m spacing near major sources, and 1 km spacing elsewhere). For major sources (>100 tons year^−1^), emissions are modeled at the stack level; other sources are modeled at the facility-level using representative stack parameters. Receptor concentrations are interpolated using inverse distance weighting to a 25 m raster that covers the study area, and block-level concentrations are estimated as areal averages of overlapping raster cells. Concentrations are predicted at the hourly level and averaged to the daily and annual periods used by the health impact functions. For NO_2_, we assume that all NO_x_ is converted to NO_2_, which may overestimate NO_2_ exposures very close to major roads.

For SO_2_, point source emissions account for nearly all emissions in the study area [45,48], and background levels are low. We use FRESH-EST to estimate daily SO_2_ exposures in 2012, and monitoring data are used to understand the extent to which FRESH-EST correctly predicts this pollutant. The health impact functions use baseline health rates that do not vary temporally; therefore, the primary concern is whether the modeled SO_2_ data represent the distribution of measured concentrations well, not if they have perfect temporal concordance. Distributions of estimated and observed daily mean SO_2_ concentrations at the Southwestern High School (SWHS, which triggered the non-attainment status for a portion of southeastern Michigan [22]) and the four closest FRESH-EST receptors (all within 200 m of the monitoring site) show no statistically significant differences (Kolmogorov-Smirnov test, all *p* values > 0.05; Figure S3). While the highest concentrations (over 85th percentile, Figure S3) measured at the SWHS monitor are under-predicted, overall, the modeled results provide acceptable estimates of SO_2_ exposures for the study population, a conclusion based on non-significant differences between the distributions of modeled and measured concentrations.

Mobile source contributions to PM_2.5_, NO_x_, and diesel particulate matter (DPM) are estimated using the RLINE dispersion model [49], a detailed link-based emission inventory for Detroit developed using the MOVES emissions model [50], 6900 receptors, and hourly meteorology. Due to the computational burden, every 6th day in 2012 is modeled. We use the same areal averaging methods from the FRESH-EST framework to estimate daily average block-level concentrations [46]. As for point sources, complete conversion of NO_x_ to NO_2_ is assumed, which may overestimate NO_2_ near major roads.

### 2.4. Apportionment of Exposures to Source Categories

Exposures are apportioned into regional, local, point, mobile, and area source categories. Point and mobile source exposures, which are estimated using dispersion modeling described earlier, and area source exposures are spatially resolved.

Exposures due to “regional” sources, representing long-range transport and secondary formation of PM_2.5_, NO_2_, and O_3_, are based on monitoring data, and all blocks are assigned the same daily regional concentration. For PM_2.5_ and NO_2_, the daily “regional” component of exposure is defined as the second lowest concentration in the monitoring network on that day. The second lowest concentration is usually similar to the lowest, but it avoids possible anomalies associated with erroneous or unrepresentative measurements. For O_3_, a secondary pollutant without direct primary emissions, the “regional” exposure is the average across all monitors in the area. This is supported by ambient monitoring that typically shows only modest changes in O_3_ levels across the study area.

“Local” exposures of PM_2.5_ and NO_2_, representing the fraction of these pollutants that come from local sources, including point, mobile, area, and secondary formation, are estimated from monitoring data. The “local increment” is estimated as the highest daily mean across the monitoring network minus the “regional” estimate. For PM_2.5_, the local increment is spatially resolved by assigning near road blocks (within 200 m of a major freeway) the full local increment; this accounts for local PM_2.5_ emissions not included in the dispersion model (e.g., secondary formation or dust) that are higher in the near-road environment; more distant blocks are assigned half of the increment. This approach is justified by the current emissions inventory, which shows that mobile sources account for approximately 50% of the PM_2.5_ emissions in Detroit [48], and by receptor modeling results that show 15% to 30% of PM_2.5_ is due to diesel exhaust and other mobile sources [51].

Estimates of area sources are included in the emissions inventory, but these lack spatial and temporal resolution, and uncertainties may be high, especially for fugitive dust. Rather than model area sources based on these uncertain emissions inventories, we estimate “area” exposures as the “local” source exposures minus the point and mobile source exposures at each census block. Any local exposures not accounted for by the point and mobile source dispersion models are captured in the “area” exposures.

A complete dataset is obtained using days for which both monitoring and modeling results are available. This results in 48 of 61 possible days modeled in 2012. Daily exposures are estimated by drawing from the distribution for complete days, and used in the health impact functions.

### 2.5. Inequality Metrics

Inequality of exposures and attributable health impact risks are evaluated at the census block. Risks are evaluated as the risk of a DALY per year, which allows impacts to be summed across health outcomes and age groups while accounting for differences in the frequency and severity of outcomes. Two inequality metrics are used. The Atkinson Index (AI), which assesses inequality across census blocks using the average health impact risk as a reference group [52], was originally developed for income inequality; more recently, it has been applied to air quality impacts [20,53,54]. The AI includes a subjective “inequality aversion” parameter, which is set to 0.75 following earlier work [20]. The second inequality metric, the concentration index (CI), evaluates how ambient concentrations and health burdens are distributed across units (e.g., individuals or census blocks) ranked by demographics or socioeconomic status [55]. Negative CI values indicate that less socially advantaged groups carry heavier burdens. Prior cumulative impact assessment work applied this metric to environmental hazards, including ambient air pollutant exposures [6,7,12,56].

The spatially-resolved demographic and vulnerability measures used by the CI are drawn from block group-level data in the 2014 five-year American Community Survey [26], specifically: percentages of the population that are non-white, identify as Hispanic or Latino, are persons of color, are foreign born, and with less than a high school diploma; percentage of households with past year income below the poverty level; and median household income (in inflation-adjusted 2014 dollars). The block-group SES variables are downscaled to the block level. (These are mapped in the Supplementary Materials.)

The sensitivity of the inequality analysis results to study boundaries and spatial resolution is examined using additional analyses. The full study domain (493 km^2^, 945,000 persons) is compared to a subdomain in southwest Detroit (79.5 km^2^, 131,000 persons, Figure 1), selected as it contains a large number of major point sources and heavily trafficked roads; this area also has been designated as non-attainment of the SO_2_ standard. Both the original study area and the subdomain are within the modeling domain for the point and mobile source dispersion models. For spatial resolution, health and inequality impacts at block- and ZIP code-level are compared. ZIP codes are selected as the unit of comparison in the sensitivity analysis because they are the smallest unit for which health data are available.

## 3. Results

### 3.1. Daily Population Exposures at the Census Block Level

NO_2_ and O_3_ concentrations show the expected seasonal variation, e.g., daily NO_2_ concentrations peak in winter and daily 8-h maximum concentrations of O_3_ peak in summer, while daily PM_2.5_ levels remain relatively consistent. Long term trends in average concentrations (2011–2015) are not apparent based on linear regression of the daily metrics (Figure S4).

Daily PM_2.5_ exposures are dominated by regional sources, which contributed an average of 8.3 μg/m^3^ across the study area, compared to 2.9 μg/m^3^ for point, mobile and area sources combined (Table 1). DPM accounts for most (90%) PM_2.5_ from on-road mobile sources. For PM_2.5_ and NO_2_, average concentrations from on-road mobile sources (0.6 μg/m^3^ and 10.2 ppb, respectively) exceed those from point sources (0.5 μg/m^3^ and 1.4 ppb, respectively). On-road mobile sources account for an average of 42% of NO_2_ exposures at the block level. In contrast to PM_2.5_, NO_2_ and O_3_, only point sources contribute to SO_2_ exposures.

### 3.2. Burden of Disease

Exposures to O_3_, PM_2.5_, SO_2_, and NO_2_ result in just over 10,000 DALYs per year incurred by residents of Detroit and the adjacent cities; this represents over $6.5 billion annually in monetized impacts (Table 2). The fraction of mortalities and morbidities attributable to air pollutant exposures varies by outcome. We estimate that 5.5% and 1.5% of annual deaths are attributable to PM_2.5_ and O_3_ exposures, respectively, which is comparable to previous estimates of attributable health burdens in the USA [19]. For morbidities, attributable fractions range from 1.6% of cardiovascular disease hospitalizations to 37% of ED visits for asthma. The sum of regional, point, mobile, and area source impacts is about 6% lower than impacts for (total) exposure of PM_2.5_ and NO_2_ due to nonlinearities in the health impact functions. Most of the health burden is due to premature mortality caused by O_3_ and PM_2.5_ exposures, specifically, 140 and 520 deaths per year among adults over 29 years of age, respectively. The most frequent attributable outcomes are minor restricted activity days (760,000 per year), missed school days (570,000 per year), and work loss days (59,000 per year); these impacts are also driven by O_3_ and PM_2.5_ exposure. Asthma exacerbations among children, which are linked to all four pollutants, are also common. Air pollutant exposures account for 3300 emergency department (ED) visits for asthma each year, largely driven by associations with O_3_ and NO_2_. PM_2.5_ and O_3_ account for most of the attributable cases of the health outcome examined. The exceptions are COPD hospitalizations and days with one or more asthma symptoms, which are driven by NO_2_. The burden attributed to mobile sources, which exceeds that of point sources, is driven by premature mortality from PM_2.5_ and asthma-related health impacts from both NO_2_ and PM_2.5_.

The excess cancer risk from DPM exposure averages 417 (SD = 199) per 10^6^ per year, and ranges from 0 to 1500 per 10^6^ per year at the block level. Our results are based on an average DPM concentration of 0.5 μg/m^3^ (range: 0–2.6 μg/m^3^) across all census blocks in the study area. Similar results have been reported in California: for a state-wide average DPM concentration of 0.58 μg/m^3^ in 2012, the excess cancer risk was 520 per 10^6^ residents per year [39]. Excess cancer risks are highest in downtown and southwest Detroit where DPM concentrations are highest (Figure S5).

### 3.3. Spatial Distribution and Inequality of Exposures and Attributable Health Burden

**Figure 2:**
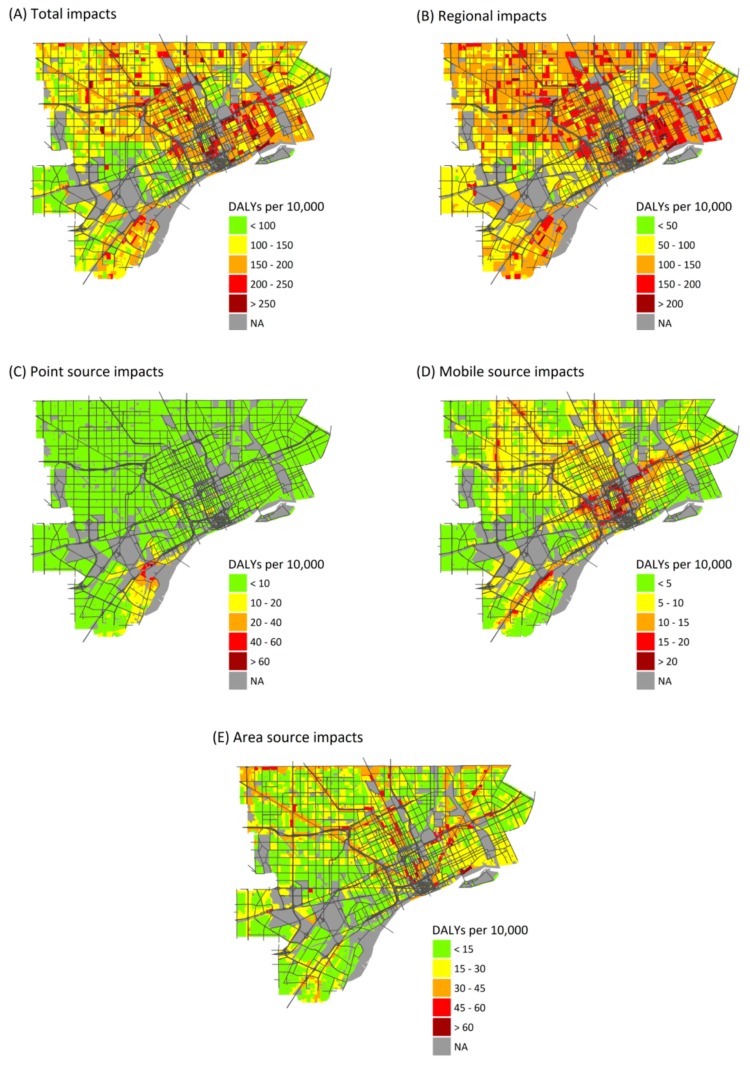
Maps showing the annual health burden as DALYs per 10,000 persons per year attributable to exposures from: all sources (**A**); and exposures from: regional sources (**B**); point sources (**C**); mobile sources (**D**); and area sources (**E**).

Health burdens attributable to air pollution exposure are unevenly distributed across the study area. Regional sources show the least variation in health burdens (Figure 2), as expected, and variation is entirely due to differences in at-risk populations and baseline health rates. (Regional PM_2.5_, O_3_, and NO_2_ exposures are assumed to be homogeneous across the study area.) Point source emissions show the heaviest burdens in central and southwest Detroit, reflecting the dispersal of emissions from point sources, most of which are located in southwest Detroit (Figure 2C). Mobile sources make their largest impacts near major roadways, especially interstate highways with a large fraction of heavy duty diesel trucks, reflecting the sharp gradients in concentrations near roads (Figure 2D) (maps of health impacts due to individual pollutants are included in Supplementary Materials).

### 3.4. Atkinson Index

Table 3 contrasts AI values for exposure concentrations (left) and health risks (right); these quantify the spatial variation seen in Figure 2. Inequality in exposure concentrations measured by the AI (0.003 to 0.130) is lower than those for health impact risks (0.040 to 0.245). Inequality is lowest for total exposures to PM_2.5_ (AI = 0.003) and NO_2_ (AI = 0.009) because these pollutants are dominated by regional sources that produce similar exposures across the study area (Table 2). In contrast, AI values for PM_2.5_, NO_2_, and SO_2_ from point and mobile sources are higher (e.g., point source exposure AIs are 0.101, 0.034 and 0.064 for PM_2.5_, NO_2_ and SO_2_, respectively; mobile source exposure AIs are 0.079 and 0.084 for PM_2.5_ and NO_2_, respectively) because the dispersion models represent spatial variability and small-scale variation. These results demonstrate the importance of using methods that account for exposure variability at the intra-urban scale; otherwise, key factors that influence vulnerability may be missed.

The inequality of health risks is especially apparent for some pollutants and sources, e.g., PM_2.5_ (AI = 0.126 and 0.126 for point and mobile sources, respectively), NO_2_ (AI = 0.159 and 0.191, respectively), and point sources of SO_2_ (AI = 0.155). AI values for the health risks (0.040 to 0.245) are considerably higher than those for exposures (0.003 to 0.130), because they account for spatial variability in exposures and baseline health risks and temporal variability in exposures. Temporal variability in exposures, which affect health impact estimates, is not well captured by averaging exposure concentrations over the full year. Including spatial variability in health risks and temporal variability in exposures is important for capturing the distribution of burdens across the population; similar exposures, whether daily or averaged over a year, in two areas with differing baseline health risks will result in unequal health burdens that are not represented by exposures alone. This contrast between inequality in exposures and health risks is especially evident for total exposures to PM_2.5_ and NO_2_. For point and mobile sources of these pollutants, the AI values for health risks are 1.24 and 4.6 times higher than the AI for exposures; for total exposures, the AI values are 13 and 15 times higher. For NO_2_, accounting for the distribution of baseline health risks across the population increases the AI from 0.009 for exposures to 0.137 for health risks. Without accounting for the underlying susceptibility of the population at the intra-urban scale, inequality in attributable health burdens due to these pollutants is underestimated, which may lead to incorrect conclusions about which pollutants result in the highest degree of inequality across the population.

**Figure 3:**
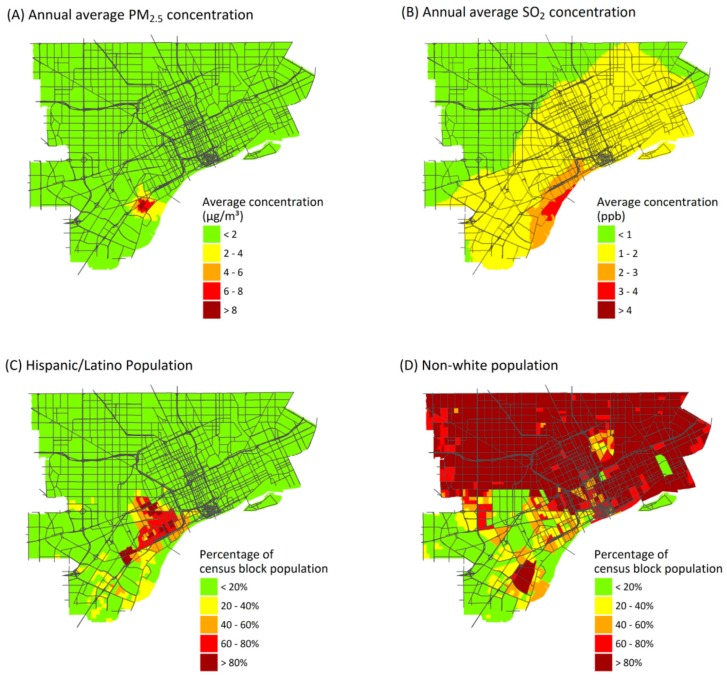
Annual average ambient concentrations from point sources of: PM_2.5_ (**A**); and SO_2_ (**B**). Percentage of the population identifying as: Hispanic or Latino (**C**); or as persons of color (excludes non-Hispanic whites) (**D**).

The AI results depend on spatial resolution. While results depend on pollutant and source, AI values for exposures and health risks at the ZIP code level averaged 17% and 47% lower, respectively, than values at the census-block level (Table 3). Larger spatial units are less likely to capture gradients in exposures and other risk factors that can increase contrasts. Interestingly, the AI value increased in one case: point source emissions of PM_2.5_. This is because the highest annual average point source concentrations of PM_2.5_ are found in a relatively small area near the sources (Figure 3A), and this variation still is represented at the ZIP code level. Although AI values tend to be lower at the ZIP code level, many of the inequality patterns remain.

The AI results are sensitive to both region and pollutant. In the sub-region defined by the SO_2_ non-attainment area, effects on AI values for health risks vary by pollutant and source, but values tend to decrease (Table 3). For example, AI values for SO_2_ decrease by 33% and 25% for exposures and health risk, respectively, in the sub-region. Most of the excluded area has low SO_2_ exposure, but some highly burdened areas remain (Figure S6C). In contrast, AI values increase for point and mobile sources of PM_2.5_ because the sub-region contains blocks with low burdens from these sources (Figure 2C) and excludes more highly burdened groups in Detroit. These analyses suggest the need for small spatial units (i.e., census blocks) that can capture exposure gradients, and study areas large enough to capture the full distribution of health impacts.

### 3.5. Concentration Index

CIs vary by pollutant, source type, and demographic or SES characteristics (Table 4), reflecting the spatial variability of exposures, health impacts, and population subgroups. (CI for pollutant exposures is shown in Table S3). The most negative values, indicating the greatest inequality, occur for point source emissions when blocks are ranked by the percentage of residents who identify as Hispanic or Latino (CI × 100 for health risks from point source emissions of PM_2.5_, SO_2_ and NO_2_ are −11.7, −13.3 and −9.3, respectively). Other variables with high CI values include percentage of the population with less than a high school diploma, and the percentage foreign born, which are moderately correlated (r = 0.42 and 0.47) to the Hispanic or Latino percentage (Figure S7). Many Hispanic and Latino residents live near locations where point sources make major impacts (Figure 3C). However, disproportionate impacts are obscured when Hispanic or Latino residents are grouped together with other minority groups in a single “persons of color” variable, which makes up most of the study population (Figure 3D), giving lower contrasts between risks for the most- and least-advantaged census blocks. Mobile source emissions also result in high CI values for rankings by income (median income and percentage of households below the poverty level). In the USA, persons earning below the poverty level are more likely to live close to major roads and thus experience higher exposures to mobile source emissions, compared to whites and more affluent groups [57,58].

The CI appears more sensitive to study boundaries than to spatial resolution (Table 4). Using ZIP code level data, increased inequality is suggested for some variables, e.g., Hispanic and Latino populations, but the same groups with the heaviest burdens are identified, suggesting that larger scale data may capture inequality effects when they are representative of population trends. However, in the study sub-region, inequality is associated with different characteristics, e.g., the percentage of the population that identifies as persons of color is associated with disproportionate burdens from point source emissions. Disproportionate impacts for non-whites can be more pronounced if the fractions of socially advantaged and disadvantaged (i.e., white and non-white) populations are more equal. Like the AI analysis, these results suggest the importance of the study boundary, the need to include exposed populations, the use of spatial units small enough to represent demographics and exposures and, in addition, whether the characteristics used to identify disproportionate groups are appropriate for the study area.

## 4. Discussion

The burden of disease and inequality assessments show that ambient air pollutant exposures can result in significant health impacts for study area residents and contribute to environmental inequalities. Five trends are highlighted for further discussion. First, exposure to air pollutants imposes a substantial health burden, even where concentrations fall below the national standards (NAAQS). Second, impacts are unevenly distributed and depend on pollutant, source type, and spatial patterns of exposure, susceptibility, and vulnerability. Third, ambient monitoring data alone are insufficient to capture the small scale variation in exposures that affect health burden and inequality analyses. Fourth, exposures as a proxy of health risks will underestimate inequality, which may hinder prioritization of strategies to alleviate health disparities. Lastly, health impact and inequality metrics depend on study boundaries and spatial resolution. These results are specific to the study area, but most findings appear applicable to other urban areas.

### 4.1. Burden of Disease Attributable to Ambient Air Pollutant Exposures below the NAAQS

The burden of disease due to pollutant exposure is significant. As noted, a portion of the study area is considered in non-attainment with the SO_2_ standard, and the entire southeast Michigan region may be designated in non-attainment with the O_3_ standard [22,23]. However, the area is in compliance with the PM_2.5_ and NO_2_ standards, and PM_2.5_ is estimated to cause most (97%) of the health burden (9800 DALYs per year, $5.1 billion per year). This estimate assumes no concentration threshold below which health effects are not expected, which is supported by recent studies showing risks below the current NAAQS, e.g., premature mortality due to PM_2.5_ [59,60]. Despite uncertainty regarding impacts of low dose exposures, continued reductions in ambient pollutant concentrations are likely to yield health benefits [61,62]. Health burden studies can help guide local, state, and national actions to further reduce concentrations, even in areas meeting current regulatory standards. Substantial benefits could be achieved by focusing on pollutants which are subject to air quality management actions in the study area. For example, NO_2_ emissions, which are an O_3_ precursor and involved in secondary PM formation [63], are likely to be targeted in a future O_3_ State Implementation Plan (SIP). Reducing NO_2_ emissions can yield large health benefits due to lower concentrations of secondary PM_2.5_ and O_3_ [18,64]. In Detroit, in addition to its role on O_3_ formation, reducing traffic emissions of NO_2_ also would reduce health inequalities associated with exposure to NO_2_ (Table 3 and Table 4).

### 4.2. Intra-Urban Inequality in the Health Burden Attributable to Ambient Air Pollution

The burden and inequality associated with point and especially mobile sources is striking, resulting in the highest health burden and disproportionately impacts on Hispanic or Latino and low income communities within the study area. This conclusion reflects the pollutant dispersion from tall stacks, as well as proximity of traffic to exposed populations. Both can cause small scale variation in pollutant concentrations, e.g., elevated concentrations of traffic-related air pollutant concentrations—and health impacts—near roadways (Figure 2D) [65,66,67], as shown in several cumulative impact studies [6,7,8]. While exposures from point sources are mostly low (Table 1), they result in disproportionate health impacts for several reasons: industry tends to cluster together (e.g., in southwest Detroit); several facilities have short stacks that cause local “hot-spots”; and predominantly Hispanic/Latino residents live nearby (Figure S2). These results depend on the spatial layout, and possibly these factors are less aligned elsewhere, e.g., where industry is farther removed from urban area, though traffic impacts are common. Site-specific studies provide perhaps the only way to understand such health burden and equity implications. Because multiple source types are implicated as having substantial health burdens on different groups, strategies aimed at addressing environmental inequalities should target multiple source types to ensure that disadvantaged communities benefit from exposure reductions.

The present analysis shows that several subgroups suffer disproportionate health impacts, e.g., Hispanic/Latino residents and low-income residents. Typically, EJ and cumulative impact analyses use a single variable (persons of color) to assess inequality by race and ethnicity (e.g., [12]). Across the larger metropolitan area (i.e., the tri-county metropolitan region or seven-county southeast Michigan region), aggregated variables may be sufficient to capture inequalities given the region’s broad patterns of racial/ethnic segregation (e.g., [25]); however, more targeted intra-urban analyses require further disaggregation by racial/ethnic minorities, otherwise, important inequalities may be missed. Characteristics used in an inequality assessment should reflect key demographic characteristics important to the specific area and should represent a reasonable fraction of the population to avoid artificially increasing inequality metrics. Addressing inequalities in exposure or risk for very small subgroups may not be feasible through public policy. Although establishing a uniform threshold for the size of the subgroup is impractical, chosen characteristics should start by identifying characteristics that highlight historical patterns of racial and ethnic segregation [68] and socioeconomic status that influence heath disparities. Within the study area and in particular within the city of Detroit (the largest city within the study area), there is less spatial variability in race (white vs. non-white) than ethnicity (Hispanic/Latino vs. non-Hispanic) and poverty status (Figure S2), and ethnicity and poverty status better capture demographic gradients within the study boundaries. Inequality analyses should also recognize that not all socially disadvantaged groups can be identified using available data. For example, in the city of Dearborn, 30% of the population identifies as Arab or Arab American [69], and many experience high exposures to social stressors [70,71]. However, the USA Census does not include data on Arab ethnicity, so this group cannot be examined with respect to inequality in health burden. In some cases, proxy characteristics, e.g., the percentage of the population that is foreign born, can be used instead (Figure S2).

The identification of disproportionately impacted populations depends in part on which populations are included and excluded from the analysis. As discussed earlier, the study boundary for this analysis is based on the area in and around Detroit expected to have the highest potential for health impacts and is limited in large part by the modeling domain used in the exposure assessment. Focusing on the entire Detroit Metropolitan area, which includes Wayne, Oakland, and Macomb counties, would likely change the interpretation of the inequality metrics and demonstrate disproportionate impacts among more groups known to be socially disadvantaged, e.g., non-Hispanic Black/African American residents. In addition, as discussed earlier, the study area has higher proportions of persons of color (75.6%) compared to Wayne County as a whole (50.2%) [26]. Compared with the study area, Oakland and Macomb counties have lower proportions of persons of color (26.0% and 17.9%, respectively) and these counties are more affluent (10.4% and 12.8% of families live below the poverty level, respectively) [26]. Residents in these counties tend to be healthier than those living in Wayne County. For example, in 2014, Oakland and Macomb counties ranked 22nd and 39th in overall health, respectively, among counties in Michigan; Wayne County consistently ranks 82nd out of 82 counties in the state [72]. Due to their distance from major sources and the presence of fewer heavily trafficked roads in Oakland and Macomb counties, we expect exposures, and thus health impacts, in these counties to be lower than the study area. Given the clustering of minority and low-income populations in the study area, the inequality metrics for exposures and health burdens are expected to be large for most if not all of the subgroups included in this study when examined within the context of the broader tri-county area.

Caution is needed when interpreting the results of the inequality assessment, which are primarily intended to demonstrate inequality between census blocks within the study area, and not necessarily the larger southeast Michigan region. In particular, the absence of evidence of a disproportionate impact for certain groups, e.g., among census blocks with high proportions of Black or African American residents, should not be interpreted as evidence that these residents are not disproportionately burdened by environmental exposures. A previous study of disparities across the tri-county region (using census tracts as the spatial unit of analysis) demonstrated residents in Detroit experience higher cumulative impacts from hazardous land uses, air pollutant exposures, and social vulnerabilities relative to other neighborhoods in the region [25]. The present analysis, which has a smaller geographical scope, is intended to help decisions makers identify those sections within the study area that are most heavily impacted, information which can be used to prioritize sections within a city for air quality management (AQM) activities. The results of this or any intra-urban inequality assessment should be interpreted within the broader body of evidence on environmental justice issues.

### 4.3. Exposures as a Poor Proxy for Health Risks in Urban-Scale Inequality Assessments

The finding from the inequality assessment that exposures alone are insufficient for representing health inequalities is important. Inequality metrics for health risks are driven, in addition to exposures, by the variability in baseline health risks, demographic variables, income, and other characteristics that influence vulnerability or susceptibility. This differs from many or possibly most earlier EJ and cumulative impact analyses that have relied on exposure indicators for a showing of disproportionate impacts, e.g., using ambient monitoring and dispersion modeling (e.g., [6,7,8,73,74,75,76]), and surrogates such as proximity to traffic or point sources [77,78]. In addition, data from national datasets like the National Air Toxics Assessment [79] and air quality monitoring networks may not have the needed spatial resolution for local scale analyses of industry and traffic pollutants [10,11]. Factors that influence the necessary resolution, including the proximity of the source to exposed populations, source characteristics such as stack heights, meteorology, the vulnerability or susceptibility of exposed populations, and other factors [80], can become more important at smaller study scales.

The choice of health impact metric for assessing inequality can be important for EJ and cumulative impact analyses. Prior analyses of attributable health impacts have focused on the risk of specific health outcomes, e.g., premature mortality or hospitalizations, and on total risk rather than attributable risk [20,53,54]. However, using a limited number of outcomes only captures a portion of the health burden due to pollutant exposures. Minor outcomes, such as asthma exacerbations or restricted activity days that contribute to overall health burdens (Table 2), should also be considered, especially if incidence rates vary spatially across the study area. DALYs provide a composite measure of health impacts that accounts for their severity and frequency and thus may be advantageous in inequality analyses. DALYs can help clarify which exposures and which sources are most important from a public health perspective. In some cumulative impact and EJ analyses, attributable risk may be more appropriate than total health risk (e.g., based on incidence rates). For example, transportation planners may be most interested in inequality in risk of mortality attributable to traffic emissions rather than total mortality risk. Although there is some uncertainty around the disability weights assigned to outcomes for DALYs [81,82], using DALYs to weight health impacts may be preferred over other cumulative impact approaches that assign uncertain weights to environmental and social determinants of health, e.g., pollutant exposures [12].

Quantitative HIA methods capture some of the burden of disease, but cannot account for other health risks for which reliable CR coefficients are not available [83] or for other non-health dimensions of environmental justice, e.g., the perception that communities are more polluted [84]. The air pollution-related outcomes for which HIFs are available are limited, and other metrics of exposure and health burden may still be important considerations. For example, proximity to environmental hazards has other important risks beyond exposure to ambient air pollutants, e.g., living near industrial facilities may negatively impact mental health [85], and noise pollution from roadways may contribute to sleep disturbance and cardiovascular disease [86]. Health impact and inequality analyses designed for specific public health decisions should include quantitative and qualitative descriptions of the health impacts of air pollutants. An explicit weighting system might be used to combine effects from descriptive and quantitative assessments.

### 4.4. Using Urban-Scale HIAs Incorporating Inequality Metrics in AQM Decision Making

Information about which sources and pollutants contribute most to environmental inequalities is important for public health priority setting, particularly at the local level where resources might be constrained. Quantitative estimates of health impacts may be particularly useful for decision makers, especially when compared to established health targets or standards [87,88]. The findings that mobile source emissions have disproportionate impacts on low income residents could be used to focus urban greening projects in areas with both high exposures and high percentages of low-income residents, thus helping to alleviate health burdens and disparities. Because increased access to green space can increase property values and make urban neighborhoods more attractive, programs to increase green space in low-income neighborhoods could be coupled with programs to support existing communities to avoid potential issues of gentrification and displacement [89]. Likewise, knowing that point and mobile sources of PM_2.5_ have a disproportionate impact in southwest Detroit (Figure 2), particularly among the Hispanic and Latino populations that live in the area (Table 4), could be used to prioritize schools in southwest Detroit for installation of filters to reduce exposures. Despite the potential benefits of using inequality metrics to inform environmental decision making, we do not necessarily advocate performing equity analyses by pollutant source for all public health decisions because health impacts result from the totality of exposure. The two examples given (increasing green space and using filters in schools) pertain to reducing exposures to specific air pollutants. Since any reduction in emissions or exposures can lead to some health benefits, identifying groups that are disproportionately impacted allows AQM activities to be targeted to simultaneously reduce health impacts and health disparities. For decisions that might increase emissions or exposures for some subpopulations, however, inequality assessments alone are not sufficient. Decisions related to permitting or facility siting, for example, should be informed from health and cumulative impact assessments that consider the total exposure to social and environmental hazards, thus avoiding unacceptable increases in the cumulative burden for any segment of the population.

Several additional considerations for urban-scale HIAs follow from the results of this study. First, exposure assessments should be based on the spatially- and temporally-resolved data that best reflect variability over the urban area. For example, several pollutants demonstrate high degrees of spatial and temporal variability that lead to greater exposures for subsections of the study area (Figure 3A,B and Figure S4), and assessing exposures using larger spatial units (e.g., ZIP codes) smooths exposure gradients across the study area (Table 3). Dispersion or land use regression models, though time- and resource-intensive, may provide a reasonable option to estimate exposures with the necessary spatial resolution [65,90,91,92]. Partnering with state or local agencies or academic partners could be helpful in building capacity to model pollutant exposures at the local level. In general, studies that utilize spatially-resolved estimates of exposure and health risks are preferred over less refined exposure assessments, and studies that incorporate health burdens in the assessment are preferred overall.

Second, the study scale and spatial resolution should be appropriate for the policy context. The sensitivity of the inequality determinations to study boundaries and spatial scale, while not surprising, emphasizes the need to structure analyses based on potential impacts and the policy or intervention context [52]. For this burden of disease assessment, the study boundaries are based on the potential for exposures and health impacts, on the ability to model these exposures and health impacts at a sufficiently fine spatial scale, and on the decision-making authority of local governments. As noted above, the results of this study could be used by decision makers with authority within the study area to prioritize AQM activities in their respective cities. The same study boundaries may not be appropriate for all intervention analyses. For assessments of specific AQM policies or programs, the selection of study boundaries needs to be deliberate and explicitly stated. For example, a decision about routing traffic, which could be influenced by a local government, requires a finely grained assessment at the intra-urban scale where impacts are expected to be localized to the area around the roadways; regional-scale decisions about how to reduce O_3_ concentrations, which involve multiple actors and require coordination across governmental agencies, would use less spatially resolved data and a larger study area. The study scale and study resolution in particular will depend on the availability of input health and exposure data. Sensitivity analyses should be used to explore implications and the robustness of selected boundary and spatial unit.

Third, the characteristics used to identify inequalities might be tailored to the specific study area. Every urban area will have a unique spatial distribution of populations, and the selection of appropriate proxies for vulnerability will vary. Variables used to examine inequality should be selected after reviewing historical and current population trends, and limitations in available data should be made explicit to decision makers and other stakeholders. For example, the Dearborn, MI area contains a large Arab and Arab American population that is not well represented using census data. However, there are large proportions of residents that are foreign born in Dearborn (Figure S2). Though not all of these foreign-born residents will be Arab or Arab American, this variable may be a reasonable proxy for the Arab population within the study area. More robust demographic datasets could potentially identify additional overburdened populations.

Lastly, HIAs using inequality metrics in a decision-making context should consider the entire policy context, not just metrics of health and inequality. It is important to note that health and inequality metrics alone do not identify optimal strategies or prioritize pollutants or source categories. Similarly, the inequality metrics provide relative measures, and there are no thresholds or standards for inequality or equality. For example, this analysis suggests PM_2.5_ from regional sources has the highest public health burden but the lowest degree of inequality, while point source emissions impose a relatively low burden but significant degree of inequality. Whether strategies should focus on achieving the greatest overall reductions in health burden or health inequality is a matter of policy. In addition to health and equity, decision makers will need to consider legal, economic, and political ramifications of public policy decisions, as well as community preferences for air quality management strategies.

### 4.5. Uncertainty in the Quantitative Health Impact Assessments

Uncertainties in quantitative HIA methods and inequality assessments influence the interpretation of results. The exposure assessment omits time-activity data, which may underestimate exposure when people spend substantial time in areas with higher concentrations than their residences [93,94]. Uncertainty around the CR coefficient has the largest influence on health impact estimates [95]. This study presents only the mean, i.e., expected, health impacts. Other sources of uncertainty include: the appropriateness and generalizability of the CR coefficient; whether the form of the HIF is appropriate; whether the exposure-outcome relationships are reasonable; the downscaling of census block group and ZIP code level demographic and baseline health rate data to the census block scale; the disability weights and duration variables used in the calculation of DALYs; uncertainties in the modeled estimates of ambient pollutant concentrations; and, potential double-counting of impacts when estimating attributable burdens from multiple pollutants [46,82,96,97,98,99]. Despite these and other uncertainties, the use of HIAs and inequality metrics offers decision-makers an objective approach to indicate the nature, magnitude, and distribution of health impacts.

## 5. Conclusions

This study has estimated the health burden attributable to exposures of PM_2.5_, O_3_, NO_2_, and SO_2_ in the Detroit area; identified the role of point, mobile, and area sources; and examined inequality of exposures and attributable health risks for population subgroups defined by demographics or socioeconomic characteristics. Exposure to ambient pollutants imposes a substantial health burden on Detroit residences, primarily due to PM_2.5_ and O_3_ exposures, most of which arises from regional sources. While local point and mobile sources impose lower health impacts overall, these sources contribute most to the inequality in the health burden experienced by socially disadvantaged populations. The methods presented can be used to inform decision making aimed at reducing environmental health burdens and inequalities, including identifying culpable sources and designing air quality management strategies to improve public health.

## Figures and Tables

**Table 1:** Summary statistics of daily concentrations of PM_2.5_ and DPM (daily mean, μg/m^3^), O_3_ (daily 8-h max, ppb), SO_2_ (daily mean, ppb), and NO_2_ (daily mean, ppb). Contributions from regional, point, mobile, and area sources are separated. Estimated at the census block level.

Pollutant	Source	Mean (SD)	Min	25th	Median	75th	95th	Max
PM_2.5_ (μg/m^3^)	Regional	8.3 (4.5)	1.5	5.2	6.8	11.3	14.5	29.5
	Point	0.5 (0.9)	0.0	0.1	0.3	0.6	1.4	75.7
	Mobile	0.6 (0.5)	0.0	0.3	0.4	0.7	1.6	12.7
	Area	1.8 (2.8)	0.0	0.2	1.0	2.2	6.3	29.4
	Total	10.7 (5.4)	2.0	6.5	9.9	13.5	19.7	82.4
DPM (μg/m^3^)	Mobile	0.5 (0.6)	0.0	0.2	0.4	0.6	1.5	12.3
O_3_ (ppb)	Regional	38.3 (13.7)	6.8	28.2	36.4	46.9	63.4	103.8
SO_2_ (ppb)	Point	1.1 (1.4)	0.0	0.1	0.5	1.6	4.0	19.4
NO_2_ (ppb)	Regional	10.9 (5.1)	2.6	7.7	9.7	12.9	23.0	30.2
	Point	1.4 (1.1)	0.0	0.5	1.1	1.9	3.5	17.0
	Mobile	10.2 (9.0)	0.0	4.3	7.6	13.0	27.1	191.9
	Area	1.7 (3.0)	0.0	0.0	0.0	2.6	8.8	17.2
	Total	23.5 (10.5)	5.8	17.3	21.9	26.0	43.1	214.2

**Table 2:** Estimated annual incidence for the health outcomes of interest and total annual burden of disease as attributable cases, disability-adjusted life years, and monetized impacts attributable to PM_2.5_, O_3_, SO_2_, and NO_2_ from regional, point, mobile, and area sources. Rounded to two significant figures.

		Attributable Impacts								
			Exposure Source				% of Attributable Burden Due to Each Pollutant			
Outcome (age group)	Estimated annual incidence ^1^	Total (% ^2^)	Regional	Point	Mobile	Area	PM_2.5_	O_3_	SO_2_	NO_2_
Mortality (cases)										
All-cause (>29)	9400	520 (5.5)	420	24	27	84	100	0	0	0
Non-accidental (>29)	8800	140 (1.5)	140	0	0	0	0	100	0	0
Infant (<1)	200	6 (4.0)	5	0	0	1	100	0	0	0
Hospitalizations (cases)										
Asthma (<65)	3200	210 (6.7)	140	17	46	16	51	0	3	46
COPD (>65)	1900	419 (22.4)	330	48	40	12	5	62	10	23
CVD (>65)	9800	160 (1.6)	130	7	8	8	100	0	0	0
Pneumonia (>65)	1500	250 (17.3)	240	3	3	3	23	77	0	0
Non-fatal MI (>17)	2600	60 (2.3)	48	3	3	3	100	0	0	0
Asthma outcomes (cases)										
Asthma ED visit (<18)	9000	3300 (36.7)	2600	160	450	120	15	51	2	31
Day w/cough (6–14)	1,700,000	210,000 (12.5)	170,000	10,000	11,000	9500	100	0	0	0
Day w/wheeze (6–14)	1,100,000	17,000 (1.6)	13,000	780	820	740	100	0	0	0
Day w/SoB (6–14)	1,000,000	21,000 (2.1)	17,000	1000	1000	940	100	0	0	0
2+ symptoms (6–14)	2,000,000	180,000 (8.6)	110,000	12,000	45,000	9600	0	34	3	64
Restricted days										
MRAD (18–64)	4,600,000	760,000 (16.7)	700,000	16,000	18,000	18,000	44	56	0	0
WLD (18–64)	1,300,000	59,000 (4.7)	47,000	2800	3000	3100	100	0	0	0
MSD (6–14)	2,700,000	570,000 (21.3)	570,000	0	0	0	0	100	0	0
Total DALYs (years)		10,000	8100	470	560	1600	97	1	0.06	1.3
Monetized impact ($ million)		6600	5500	240	280	830	78	21	0.03	0.5

^1^ Estimated annual incidence rates based on block group population and ZIP code level incidence rates. ^2^ Percentage of the estimated annual incidence attributable to all pollutant exposures. Abbreviations: COPD: Chronic obstructive pulmonary disease; CVD: cardiovascular disease; DALYs: disability-adjusted life years; ED: emergency department; MI: myocardial infarction; MRAD: minor restricted activity day; MSD: missed school day; SoB: shortness of breath; WLD: work loss day.

**Table 3:** Atkinson index (AI) ^1^ for annual average pollutant exposure and annual health impact (as risk of a DALY per year) attributable to individual pollutants for the full analysis, and for sensitivity analyses of spatial resolution and region. Percentages (in parentheses) show change from “all blocks”. Negative percentages indicate increases in AI.

		Annual Average Exposures ^2^			Annual Health Impact Risk		
Pollutant	Source	All Blocks	ZIP Codes	NA Area ^3^	All Blocks	ZIP Codes	NA Area ^3^
PM_2.5_	Regional ^4^	-	-	-	0.041	0.022 (46)	0.038 (7)
	Point	0.101	0.139 (−37)	0.107 (−5)	0.126	0.154 (−22)	0.157 (−25)
	Mobile	0.079	0.057 (29)	0.128 (−61)	0.126	0.084 (34)	0.153 (−21)
	Area	0.070	0.019 (73)	0.082 (−18)	0.113	0.045 (60)	0.111 (1)
	Total	0.003	0.001 (62)	0.003 (−13)	0.045	0.023 (49)	0.041 (8)
O_3_	Regional ^4^	-	-	-	0.040	0.023 (43)	0.038 (4)
SO_2_	Point	0.064	0.055 (13)	0.043 (33)	0.155	0.075 (51)	0.116 (25)
NO_2_	Regional ^4^	-	-	-	0.133	0.038 (72)	0.096 (28)
	Point	0.034	0.027 (23)	0.042 (−21)	0.159	0.057 (64)	0.140 (12)
	Mobile	0.084	0.055 (34)	0.126 (−50)	0.191	0.072 (62)	0.203 (−7)
	Area	0.130	0.101 (22)	0.163 (−26)	0.245	0.141 (43)	0.225 (8)
	Total	0.009	0.011 (−18)	0.012 (−25)	0.137	0.045 (67)	0.104 (24)

^1^ Inequality aversion parameter set to 0.75. ^2^ PM_2.5_, SO_2_, and NO_2_ are reported as the average of daily mean concentrations and O_3_ is reported as the average of daily 8-h maximum concentrations. ^3^ Subset of study area census blocks that are within the SO_2_ non-attainment area. ^4^ Regional exposures are omitted from the Atkinson index because all spatial units are assigned the same concentration. Abbreviations: NA: Non-attainment.

**Table 4:** Concentration index values (×100) for annual risk of a DALY per year attributable to individual pollutants for the full analysis, and for sensitivity analyses of spatial resolution and region. Negative values indicate disproportionately high health burdens in socially disadvantaged spatial units. Percentages (in parentheses) show change from “all blocks”.

		Concentration Index (×100)						
Pollutant	Source	Percent Non-White	Percent Latino	Percent Less than HS	Median Income	Percent HH in Poverty	Percent POC	% FB
*All census blocks*								
PM_2.5_	Regional	−6.7	3.0	−1.0	−4.1	−1.2	−6.4	6.5
	Point	5.4	−11.7	−8.2	−3.1	−0.2	3.8	−5.7
	Mobile	−6.6	0.8	−4.6	−8.7	−5.5	−6.8	6.1
	Area	−7.6	4.0	0.4	−4.5	−1.8	−7.0	7.9
	Total	−6.3	2.4	−1.3	−4.4	−1.5	−6.1	6.1
O_3_	Regional	−6.2	3.0	−0.6	−3.4	−0.5	−5.9	6.1
SO_2_	Point	8.1	−13.3	−11.1	−3.3	−6.9	6.0	−12.4
NO_2_	Regional	1.3	−2.1	−3.6	−0.8	−5.0	0.7	−4.3
	Point	5.8	−9.3	−8.9	−2.8	−7.0	4.1	−10.1
	Mobile	−1.0	−3.4	−7.2	−5.0	−8.4	−2.3	−2.6
	Area	3.6	−0.6	2.8	4.4	0.2	4.1	−4.7
	Total	0.8	−3.0	−4.9	−2.3	−6.1	−0.1	−3.9
*ZIP codes*								
PM_2.5_	Regional	−8 (−19)	4.1 (−35)	−0.5 (49)	−8.2 (−101)	−4.3 (−269)	−8.2 (−27)	7.8 (−21)
	Point	8.5 (−58)	−23.7 (−102)	−12.9 (−57)	−6.1 (−95)	−6.1 (−2973)	2.2 (41)	−8.2 (−44)
	Mobile	−4.4 (33)	−0.8 (193)	−1.4 (70)	−18.6 (−114)	−13.5 (−143)	−6.1 (11)	1.1 (81)
	Area	−9.2 (−22)	8.7 (−121)	1 (−139)	−8.1 (−81)	−4.8 (−165)	−8.6 (−23)	10.1 (−28)
	Total	−7.7 (−21)	3 (−25)	−0.6 (57)	−8.1 (−85)	−3.9 (−166)	−8 (−32)	7.4 (−21)
O_3_	Regional	−8.2 (−32)	4.6 (−52)	−0.8 (−35)	−8.4 (−149)	−4.2 (−823)	−8.1 (−39)	7.5 (−23)
SO_2_	Point	11.1 (−37)	−15.2 (−15)	−18.7 (−68)	−3.2 (4)	−7.2 (−4)	8.3 (−40)	−12.1 (3)
NO_2_	Regional	1.5 (−16)	−0.9 (58)	−6.5 (−84)	1.6 (296)	−2.2 (56)	1.1 (−59)	−3 (30)
	Point	8.5 (−46)	−11 (−19)	−15.1 (−69)	−1.1 (59)	−5.7 (18)	6.3 (−53)	−10.1 (0)
	Mobile	1.9 (295)	−2.7 (20)	−9.1 (−27)	−7.8 (−55)	−10.3 (−24)	0.2 (107)	−6.5 (−154)
	Area	−3.9 (207)	8.4 (1421)	4.8 (−72)	7.5 (−69)	4.8 (−2110)	−2.4 (158)	9.4 (301)
	Total	3.3 (−316)	−4.4 (−47)	−10.1 (−106)	0.1 (105)	−4.2 (32)	2.1 (1867)	−6.3 (−60)
*Census blocks in the SO_2_ non-attainment area*								
PM_2.5_	Regional	−3.8 (43)	6.1 (−102)	5.8 (702)	0.6 (115)	3 (362)	−1.3 (79)	7.1 (−10)
	Point	−11.3 (310)	2.7 (123)	−3.2 (61)	−8.9 (−184)	−5.5 (−2663)	−11.6 (406)	6.2 (208)
	Mobile	−9.2 (−40)	−0.9 (205)	−0.1 (98)	−6 (31)	−4.1 (26)	−9.3 (−37)	1.8 (71)
	Area	−0.4 (95)	7.8 (−98)	10.9 (−2598)	5.7 (227)	6 (430)	3.3 (147)	7.7 (2)
	Total	−4.4 (31)	5.4 (−125)	5 (486)	−0.1 (97)	2.1 (241)	−2.2 (64)	6.6 (−8)
O_3_	Regional	−3.3 (46)	6.5 (−117)	6.5 (1168)	1.5 (143)	3.9 (965)	−0.8 (87)	7.1 (−18)
SO_2_	Point	−6.1 (175)	−11.2 (16)	−12.6 (−13)	−8.7 (−160)	−10.9 (−58)	−9.2 (254)	−10.1 (19)
NO_2_	Regional	−0.6 (148)	−8.6 (−319)	−8.4 (−137)	−3.9 (−394)	−6.1 (−24)	−3.5 (609)	−9 (−109)
	Point	−5.9 (202)	−12 (−29)	−13.4 (−49)	−9.7 (−252)	−11.9 (−71)	−9.7 (334)	−11.2 (−11)
	Mobile	−5.7 (−469)	−16.8 (−392)	−15.3 (−115)	−10.2 (−103)	−12.5 (−50)	−11.1 (−375)	−15 (−488)
	Area	6.5 (−82)	7.2 (1225)	8.1 (−189)	7.6 (−73)	6.4 (−2835)	9.2 (−125)	7 (250)
	Total	−2.6 (429)	−11.3 (−279)	−10.6 (−117)	−6.1 (−170)	−8.4 (−37)	−6.2 (−5256)	−10.6 (−171)

## Supplementary Materials

The following are available online at www.mdpi.com/1660-4601/14/10/1243/s1. Table S1: Pollutants, health outcomes, age groups, and concentration-response coefficients used in the health impact functions; Table S2: Disability weights, duration, and monetary values used to estimate disability-adjusted life years and monetized impacts; Table S3, Concentration index values (×100) for annual average exposure concentration attributable to individual ambient air pollutants for the full analysis and the two sensitivity analyses; Figure S1: Maps of baseline health rates used in the health impact functions; Figure S2: Maps of SES variables used to rank census blocks when calculating the concentration index; Figure S3: Comparison of the distributions of measured daily mean SO_2_ concentrations at the Southwest High School monitor (2011–2015) and modeled FRESH-EST receptors within 150 m of the monitor; Figure S4: Daily concentrations of NO_2_ (daily mean, ppb), O_3_, (daily 8-h max, ppb), and PM_2.5_ (daily mean, μg/m^3^) averaged across monitors in the Detroit, MI area; Figure S5: Annual diesel particulate matter (DPM) concentrations ((A), μg/m^3^) and excess cancer risk ((B), excess cases per 10^6^) due to DPM exposures measured at the census block level; Figure S6: Maps showing the burden of disease (as DALYs per 10,000 per year) attributable to total exposures of (A) PM_2.5_, (B) ozone, (C) SO_2_, and (D) NO_2_; and Figure S7: Correlations between block-level demographic and socioeconomic variables in the study area.

## Appendix A

The following section provides additional details on the methods used in this study.

Each health impact function (HIF) predicts the number of attributable cases (Y) and requires four inputs: (1) a baseline incidence rate for the health outcome of interest (y_0_, cases per person per day); (2) a concentration-response relating an exposure concentration to a change in health outcome risk (β, risk per unit exposure); (3) an estimate of the exposure concentration (x, units of concentration, e.g., ppb or μg/m^3^); and (4) an estimate of the exposed population (P, persons). The HIF is derived from the expression of relative risk, and the form of the equation depends on the model used to estimate the concentration response (CR) coefficient. This study relies on two forms of the HIF: a log-linear form (A1) and a logistic form (A2).

Y = y_0_ (1 − e^−β × x^) P
(A1)

Y = y_0_ (1 − 1/{[1 – y_0_] e^β × x^ + y_0_}) P (A2)

We apply the HIF for each pollutant-outcome pair to each census block *u* using block-specific estimates of baseline rates (y_0,u_), exposure concentrations (x_u_), and exposed populations (p_u_) to estimate the number of block-specific attributable impacts (Y_u_). The total number of attributable health impacts for each pollutant-outcome pair across the entire study area is given by $\sum_{u=1}^{n}Y_{u}$, where *n* is the total number of census blocks in the study area.

The health outcomes included in the HIFs were chosen based on the strength of causal association as determined by the USA EPA in their Integrated Science Assessments (ISAs) [33,34,35,100]. Baseline incidence rates used in the HIFs come from multiple sources and are available at various spatial scales. Rates are downscaled to the census block level. Mortality rates at the ZIP code level (2009–2013) are calculated using geocoded mortality data made available by the Michigan Department of Health and Human Services (MDHHS) and 5-year age-stratified population estimates from the 2013 ACS survey [29]. Hospitalization rates at the ZIP code level (2009–2013) are based on hospitalization data for Wayne County hospitals and 2013 ACS survey population estimates. Rates for ED visits for asthma are available at the ZIP code level for Detroit and the county level outside of Detroit [101,102]. Rates for Asthma related respiratory symptom day rates are taken from a cohort study of children with asthma in Detroit [103]. Rates for other health outcomes, including non-fatal heart attacks, work and school absence days, and minor-restricted activity days are not available for the study area, so national rates used in HIA conducted by the USA EPA are substituted [104]. Maps showing the ZIP-code level baseline health rates are included below.

The CRs used in this study have been taken from studies identified by the USA EPA for inclusion in Integrated Science Assessments for O_3_, PM_2.5_, SO_2_, and NO_2_ [33,34,35,100]. The BenMAP User’s Manual [104] and the epidemiological literature were also reviewed to identify other potential studies for inclusion. In addition to the studies summarized by the ISAs, effect estimates from studies conducted in Detroit were also considered, as local studies may better reflect the underlying risk than studies conducted elsewhere, but can be subject to limitations based on statistical power or study design [105]. The CR coefficients for each of the pollutant-outcome pairs are listed in Table S1.

The HIA methods described here use three metrics to estimate health burden: the number of incident cases of mortality or morbidity attributable to pollutant exposure (attributable cases), disability-adjusted life years (DALYs), and monetized impacts. DALYs and monetized impacts are derived from the number of attributable cases. A DALY is the sum of years of life lost (due to premature mortality) and years lived with disability (due to morbidity), and calculations require a disability-weight (DW) and duration (D) for each outcome [40]. Monetized values are typically assigned to mortalities based on the value of a statistical life (VSL) and to morbidities based on the cost of illness (COI) or willingness to pay (WTP) estimates [106]. In order to monetize the health impacts, monetary values from the Regulatory Impact Analysis for the most recent particulate matter standard in the USA are used [16]. Monetized values are reported in 2010 dollars projected to a 2020 income level. DW, duration and monetized values for each of the health outcomes in the HIA are listed in Table S2.
