# Supplementary material for: Disease and Health Inequalities Attributable to Air Pollutant Exposure in Detroit, Michigan
Source: Int J Environ Res Public Health. 2017 Oct 19;14(10):1243. doi: 10.3390/ijerph14101243 (PMC5664744; doi:10.3390/ijerph14101243)
Supplement: Supplementary file 1 [file ijerph-14-01243-s001.pdf]

International Journal of
Environmental Research and Public Health

MDPI

Supplemental Materials: Disease and health inequalities attributable to air pollutant exposure in Detroit, Michigan

Sheena E. Martenies [1], Chad W. Milando [2], Guy O. Williams [3] and Stuart A. Batterman [4,*]

Supplemental Tables:

Table S1. Pollutants, health outcomes, age groups, and concentration-response coefficients used in the health impact functions

Table S2. Disability weights, duration, and monetary values used to estimate disability-adjusted life years and monetized impacts

Table S3. Concentration index values (× 100) for annual average exposure concentration attributable to individual ambient air pollutants for the full analysis and the two sensitivity analyses. Percentages in parentheses are the percent difference between the sensitivity analysis values and the "all blocks" analysis.

Supplemental Figures:

Figure S1. Maps of baseline health rates used in the health impact functions.

Figure S2. Maps of SES variables used to rank census blocks when calculating the concentration index. Figure S3. Comparison of the distributions of measured daily mean SO_2 concentrations at the Southwest High School monitor (2011–2015) and modeled FRESH-EST receptors within 150 m of the monitor. K-S tests for each receptor are all non-significant ($p > 0.05$)

Figure S4. Daily concentrations of NO_2 (daily mean, ppb), O_3, (daily 8-hour max, ppb), and $PM_{2.5}$ (daily mean, μg/m3) averaged across monitors in the Detroit, MI area.

Figure S5. Annual diesel particulate matter (DPM) concentrations (A, μg/m^3) and excess cancer risk (B, excess cases per 10^6) due to DPM exposures measured at the census block level.

Figure S6. Maps showing the burden of disease (as DALYs per 10,000 per year) attributable to total exposures of (A) $PM_{2.5}$, (B) ozone, (C) SO_2, and (D) NO_2. The sub-region of the study area that is in non-attainment of the SO_2 National Ambient Air Quality Standard is shown (blue polygon).

Figure S7. Correlations between block-level demographic and socioeconomic variables in the study area

Supplemental Tables

Table S1. Pollutants, health outcomes, age groups, and concentration-response coefficients used in the health impact functions

Pollutant	Health Outcome	Age group	CR	Form	Reference
O_3	Non-accidental mortality	30+	0.00041	log-linear	Smith et al. (2009) [1]
	ED visit for asthma	0-17	0.01044	log-linear	Mar and Koenig (2009) [2]
	Asthma symptom day (one or more symptoms)	6-14	0.00194	logistic	Schildcrout et al. (2006) [3]
	Pneumonia hospitalization	65+	0.00521	log-linear	Schwartz (1994) [4]
	COPD hospitalization	65+	0.00549	log-linear	Schwartz (1994) [4]
	Missed school day	6-14	0.00755	log-linear	Gilliland et al. (2001) [5]
	Minor restricted activity day	18-64	0.00260	log-linear	Ostro and Rothschild (1989) [6]
$PM_{2.5}$	All-cause mortality	30+	0.00545	Log-linear	Krewski et al (2009) [7]
	Infant mortality	0-1	0.00392	logistic	Woodruff et al. (1997) [8]
	Asthma hospitalization	0-64	0.00332	log-linear	Sheppard et al. (2003) [9]
	COPD hospitalization	65+	0.00117	log-linear	Ito et al. (2003) [10]
	CVD hospitalization	65+	0.00158	log-linear	Moolgavkar (2003) [11]
	Pneumonia hospitalization	65+	0.00398	log-linear	Ito et al (2003) [10]
	Non-fatal heart attack	18+	0.00222	logistic	Zanobetti et al (2008) [12]
	ED visit for asthma	0-17	0.00560	log-linear	Mar et al. (2010) [13]
	Asthma symptom day (cough)	6-14	0.01906	logistic	Mar et al. (2004) [14]
	Asthma symptom day (shortness of breath)	6-14	0.00256	logistic	Ostro et al. (2001) [15]
	Asthma symptom day (wheeze)	6-14	0.00194	logistic	Ostro et al. (2001) [15]
	Minor restricted activity day	18-64	0.00741	log-linear	Ostro and Rothschild (1989) [6]
	Work loss day	18-64	0.00460	log-linear	Ostro (1987) [16]
SO_2	Asthma hospitalization	0-64	0.00203	log-linear	Sheppard (2003) [9]
	COPD hospitalization	65+	0.02081	log-linear	Yang et al. (2005) [17]
	ED visit for asthma	0-17	0.00853	log-linear	Ito et al. (2007) [18]
	ED visit for asthma (Detroit CR)	0-17	0.00976	log-linear	Li et al. (2011) [19]
	Asthma symptom day (one or more symptoms)	6-14	0.00392	logistic	Schildcrout et al. (2006) [3]
	Asthma symptom day (one or more symptoms, Detroit CR)	6-14	0.01695	logistic	Batterman et al. (in prep) [20]
NO_2	Asthma hospitalization	0-64	0.00140	log-linear	Linn et al. (2000) [21]
	COPD hospitalization	65+	0.0024	log-linear	Moolgavkar (2003) [11]
	ED visit for asthma	0-17	0.00546	log-linear	Ito et al. (2007) [18]
	Asthma symptom day (one or more symptoms)	6-14	0.00431	logistic	Schildcrout et al. (2006) [3]

Table S2. Disability weights, duration, and monetary values used to estimate disability-adjusted life years and monetized impacts

Outcome	Age	DW (--)	D (years)	V ($)	DW Source	D Source	V Source
Mortality							
All-cause	30-34	1	49.327	9600000		MDHHS, 2015 [22]	US EPA, 2012 [23]
All-cause	35-30	1	44.645	9600000		MDHHS, 2015 [22]	US EPA, 2012 [23]
All-cause	40-44	1	39.978	9600000		MDHHS, 2015 [22]	US EPA, 2012 [23]
All-cause	45-49	1	35.406	9600000		MDHHS, 2015 [22]	US EPA, 2012 [23]
All-cause	50-54	1	30.962	9600000		MDHHS, 2015 [22]	US EPA, 2012 [23]
All-cause	55-59	1	26.726	9600000		MDHHS, 2015 [22]	US EPA, 2012 [23]
All-cause	60-64	1	22.653	9600000		MDHHS, 2015 [22]	US EPA, 2012 [23]
All-cause	65-69	1	18.745	9600000		MDHHS, 2015 [22]	US EPA, 2012 [23]
All-cause	70-74	1	15.056	9600000		MDHHS, 2015 [22]	US EPA, 2012 [23]
All-cause	75-79	1	11.68	9600000		MDHHS, 2015 [22]	US EPA, 2012 [23]
All-cause	80-84	1	8.627	9600000		MDHHS, 2015 [22]	US EPA, 2012 [23]
All-cause	85+	1	5.9	9600000		MDHHS, 2015 [22]	US EPA, 2012 [23]
Infant	0-1	1	77.923	9600000		MDHHS, 2015 [22]	US EPA, 2012 [23]
Hospitalizations							
Asthma	0-64	0.64	0.009	16000	de Hollander 1999 [24]	CDC, 2012 [25]	US EPA, 2012 [23]
COPD	65+	0.64	0.012	36000	de Hollander 1999 [24]	CDC, 2012 [25]	US EPA, 2012 [23]
CVD	65+	0.71	0.0126	41000	de Hollander 1999 [24]	CDC, 2012 [25]	US EPA, 2012 [23]
Pneumonia	65+	0.64	0.014	36000	de Hollander 1999 [24]	CDC, 2012 [25]	US EPA, 2012 [23]
Non-fatal MI	18+	0.42	0.015	143000	de Hollander 1999 [24]	CDC, 2012 [25]	US EPA, 2012 [23]
Asthma outcomes							
ED Visit	0-17	0.51	0.0027	430	de Hollander 1999 [24]		US EPA, 2012 [23]
Cough		0.22	0.005	58	de Hollander 1999 [24]		US EPA, 2012 [23]
SoB		0.22	0.005	58	de Hollander 1999 [24]		US EPA, 2012 [23]
Wheeze		0.22	0.005	58	de Hollander 1999 [24]		US EPA, 2012 [23]
One or more		0.22	0.005	58	de Hollander 1999 [24]		US EPA, 2012 [23]
Restricted activity days							
MRAD		0.092	0.0027	68	Murray, 1994 [26]	Ostro, 1987 [16]	US EPA, 2012 [23]
WLD		0.092	0.0027	150	Murray ,1994 [26]		US EPA, 2012 [23]
SLD		0.092	0.0027	98			US EPA, 2016 [27]

Abbreviations: COPD: chronic obstructive pulmonary disease; CVD: cardiovascular disease; D: duration; DW: disability weight; ED: emergency department; MI: myocardial infarction; MRAD: minor restricted activity day; SLD: school loss day (school absence); WLD: work loss day.

Table S3. Concentration index values (× 100) for annual average exposure concentration attributable to individual ambient air pollutants for the full analysis and the two sensitivity analyses. Percentages in parentheses are the percent difference between the sensitivity analysis values and the "all blocks" analysis.

Pollutant	Source	Concentration index (× 100)						
		% non-white	% Latino	% less than HS	Median income	% HH in poverty	% POC	% FB
		All census blocks						
$PM_{2.5}$	Regional	—	—	—	—	—	—	—
	Point	12.9	−15.4	−8.9	0.8	0.7	10.9	−13.2
	Mobile	−0.5	−2.6	−4.0	−4.4	−4.0	−1.1	0.0
	Area	−1.1	0.9	1.7	−0.2	−0.2	−0.8	1.7
	Total	0.4	−0.7	−0.4	−0.3	−0.2	0.3	−0.4
O_3	Regional	—	—	—	—	—	—	—
SO_2	Point	6.8	−10.6	−7.0	−2.7	−2.9	5.7	−7.8
NO_2	Regional	—	—	—	—	—	—	—
	Point	4.3	−6.6	−4.9	−2.0	−2.3	3.5	−5.2
	Mobile	−2.0	−1.0	−3.6	−4.5	−4.1	−2.6	1.4
	Area	3.4	0.6	6.4	5.9	6.1	4.2	−0.8
	Total	−0.3	−0.8	−1.3	−1.6	−1.4	−0.5	0.2
		ZIP codes						
$PM_{2.5}$	Regional	—	—	—	—	—	—	—
	Point	0.139 (−37)	16 (−23)	−27 (−76)	−14.8 (−66)	1 (−35)	−2.7 (508)	9.7 (11)
	Mobile	0.057 (29)	2.7 (681)	−4.5 (−71)	−2.2 (46)	−9.9 (−126)	−8.4 (−110)	1.2 (208)
	Area	0.019 (73)	−1.8 (−61)	4.9 (−425)	1.6 (10)	−0.1 (55)	−0.8 (−301)	−0.9 (−13)
	Total	0.001 (62)	0.5 (−17)	−1.2 (−72)	0.1 (116)	0.2 (185)	0.5 (295)	0.2 (26)
O_3	Regional	—	—	—	—	—	—	—
SO_2	Point	0.055 (13)	9.5 (−40)	−13.7 (−29)	−10.7 (−53)	−4.4 (−60)	−4.8 (−64)	7.7 (−36)
NO_2	Regional	—	—	—	—	—	—	—
	Point	0.027 (23)	6.9 (−59)	−9.8 (−48)	−7.5 (−54)	−2.6 (−31)	−3.4 (−48)	5.4 (−52)
	Mobile	0.055 (34)	1.1 (152)	−2.5 (−163)	−1.4 (60)	−9.9 (−120)	−8.5 (−109)	−0.1 (95)
	Area	0.101 (22)	−4.8 (239)	8.4 (−1278)	7.9 (−24)	4.2 (28)	4.7 (23)	−3.5 (183)
	Total	0.011 (−18)	1.6 (578)	−3.1 (−291)	−2.1 (−56)	−0.6 (60)	−0.9 (36)	1.2 (315)
		Census blocks in the SO_2 non−attainment area						
$PM_{2.5}$	Regional	—	—	—	—	—	—	—
	Point	0.107 (−5)	−6.9 (153)	−3.7 (76)	−9.5 (−7)	−9.5 (1328)	−9.1 (1482)	−9.4 (186)
	Mobile	0.128 (−61)	−4.7 (−914)	−7.5 (−186)	−6.4 (−58)	−6 (−37)	−6.3 (−57)	−7.3 (−556)
	Area	0.082 (−18)	3.7 (429)	2.2 (−134)	5.8 (−233)	5.2 (3341)	4 (2091)	5 (719)
	Total	0.003 (−13)	−0.5 (217)	−0.7 (−2)	−0.8 (−110)	−0.7 (−178)	−0.9 (−242)	−0.7 (339)
O_3	Regional	—	—	—	—	—	—	—
SO_2	Point	0.043 (33)	−6.4 (193)	−4 (62)	−5.8 (17)	−6 (−118)	−6.5 (−123)	−6.7 (219)
NO_2	Regional	—	—	—	—	—	—	—
	Point	0.042 (−21)	−6.2 (244)	−3.1 (53)	−5.4 (−12)	−6 (−199)	−6.2 (−173)	−6.7 (290)
	Mobile	0.126 (−50)	−4.8 (−133)	−7.4 (−670)	−6.6 (−82)	−6.1 (−35)	−6.4 (−58)	−7.4 (−190)

Area	0.163 (−26)	6.5 (−91)	14.3 (−2242)	15.7 (−145)	10.8 (−85)	12 (−95)	11.5 (−171)
Total	0.012 (−25)	−2 (−494)	−2.4 (−207)	−2.1 (−55)	−2.2 (−38)	−2.3 (−64)	−2.8 (−412)

Supplemental Figures

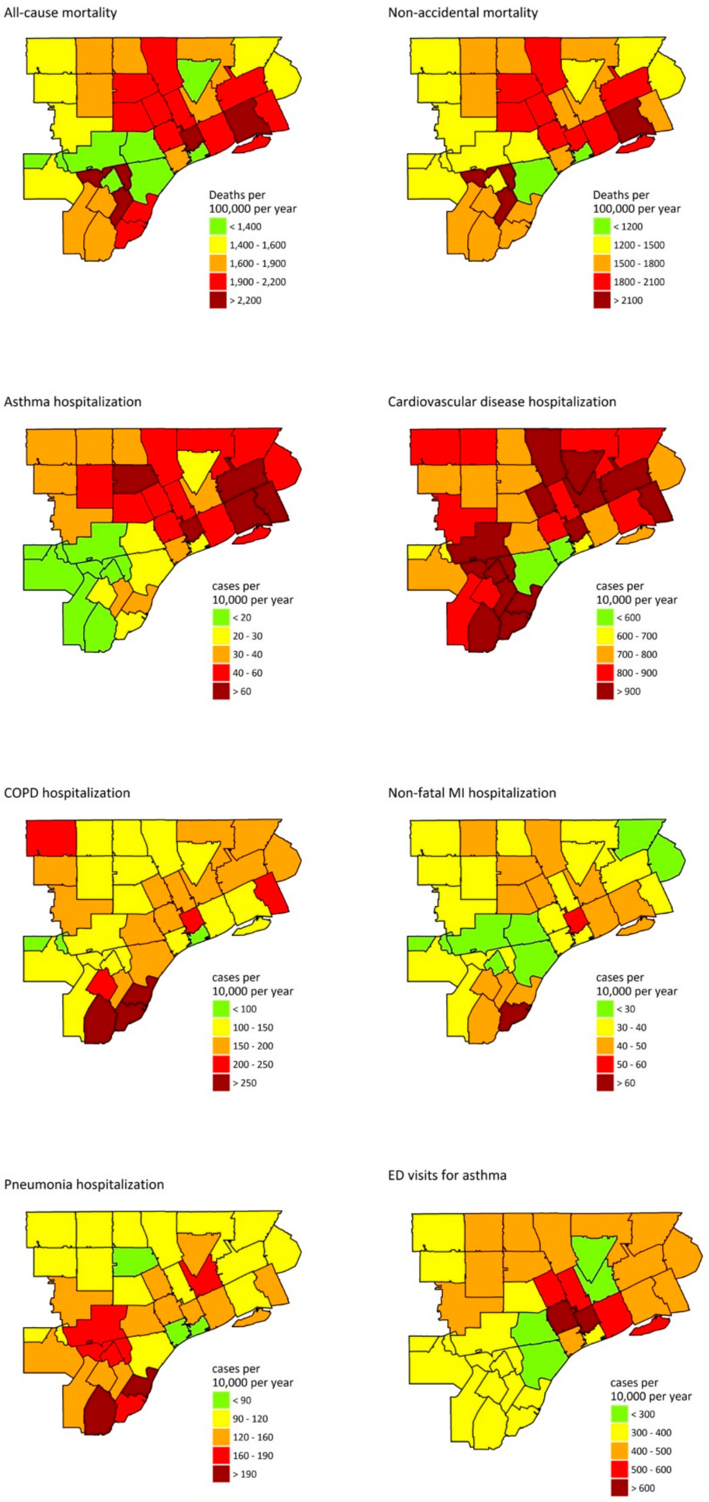

Figure S1. Maps of baseline health rates used in the health impact functions.

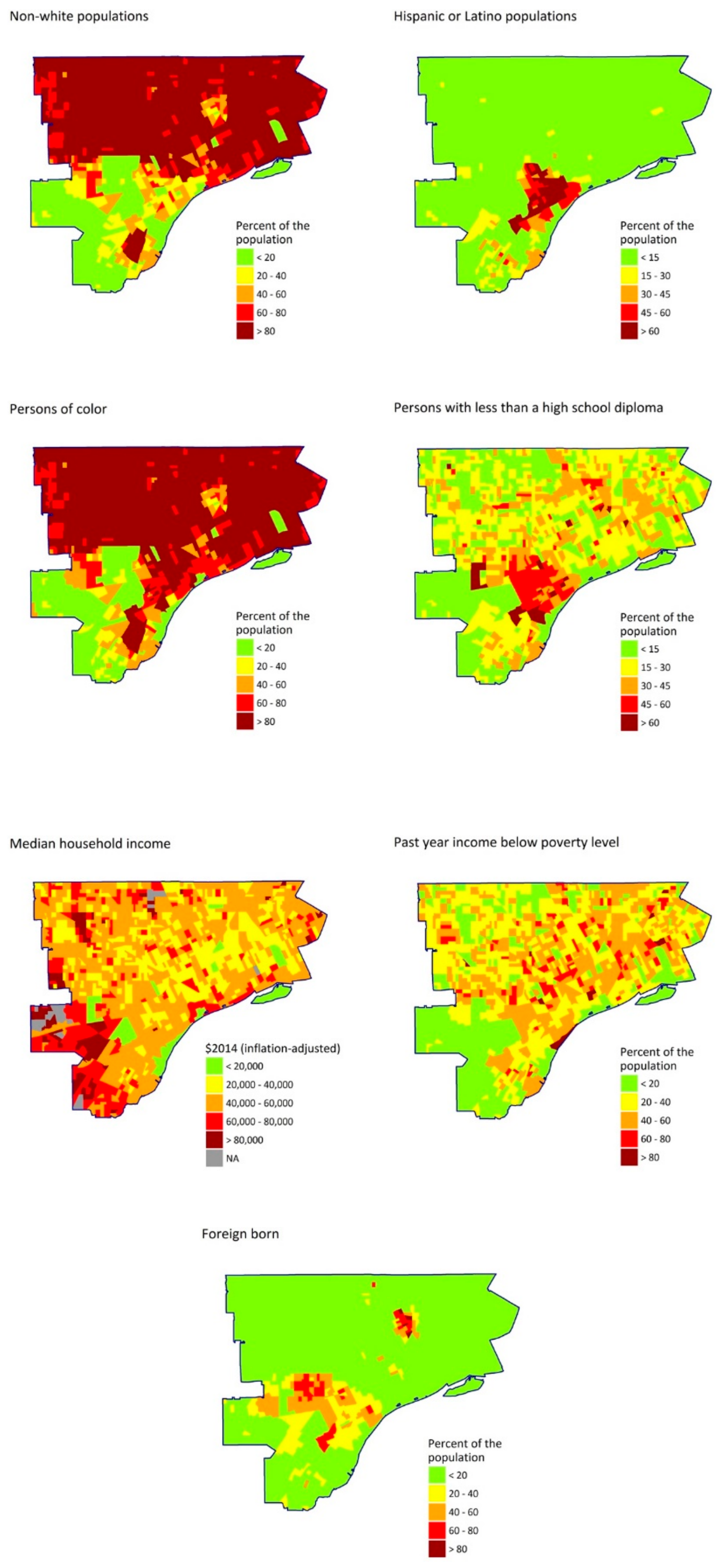

Figure S2. Maps of SES variables used to rank census blocks when calculating the concentration index.

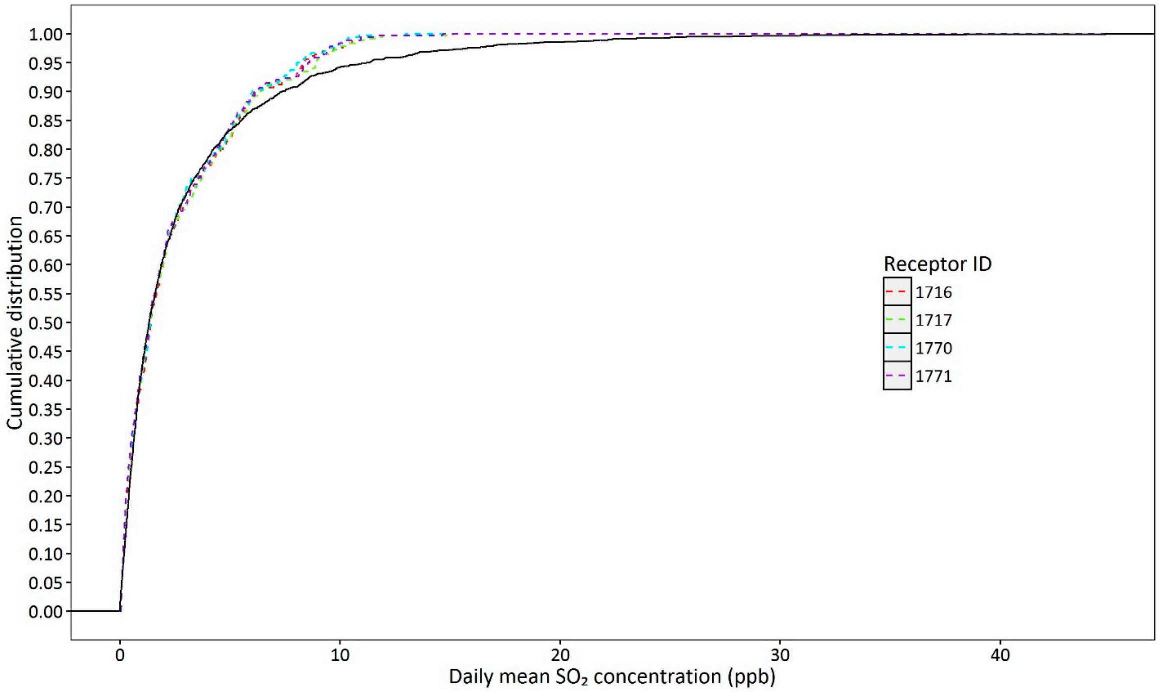

Figure S3. Comparison of the distributions of measured daily mean SO_2 concentrations at the Southwest High School monitor (2011−2015) and modeled FRESH−EST receptors within 150 m of the monitor. K−S tests for each receptor are all non−significant ($p > 0.05$)

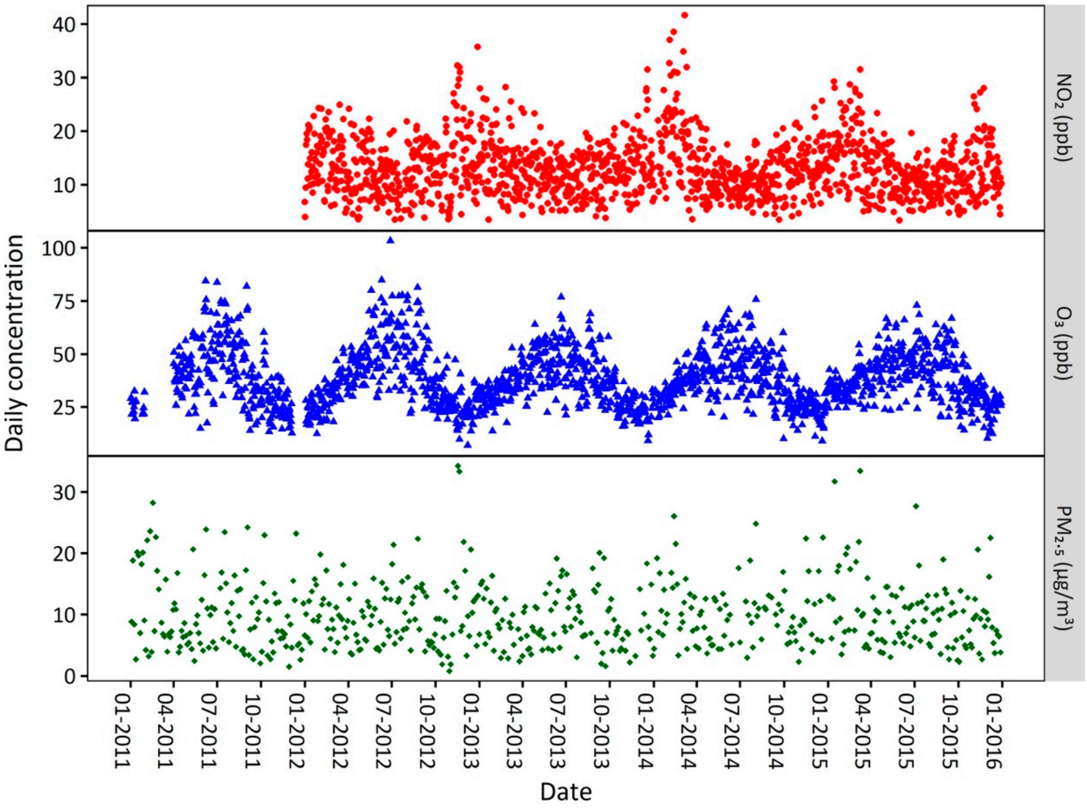

Figure S4. Daily concentrations of NO_2 (daily mean, ppb), O_3, (daily 8−hour max, ppb), and $PM_{2.5}$ (daily mean, μg/m^3) averaged across monitors in the Detroit, MI area.

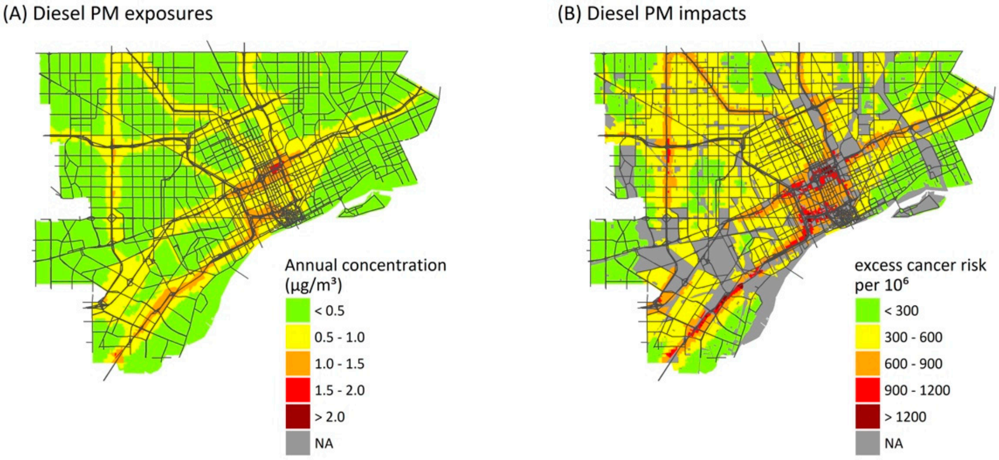

Figure S5. Annual diesel particulate matter (DPM) concentrations (**A**, μg/m^3) and excess cancer risk (**B**, excess cases per 10^6) due to DPM exposures measured at the census block level.

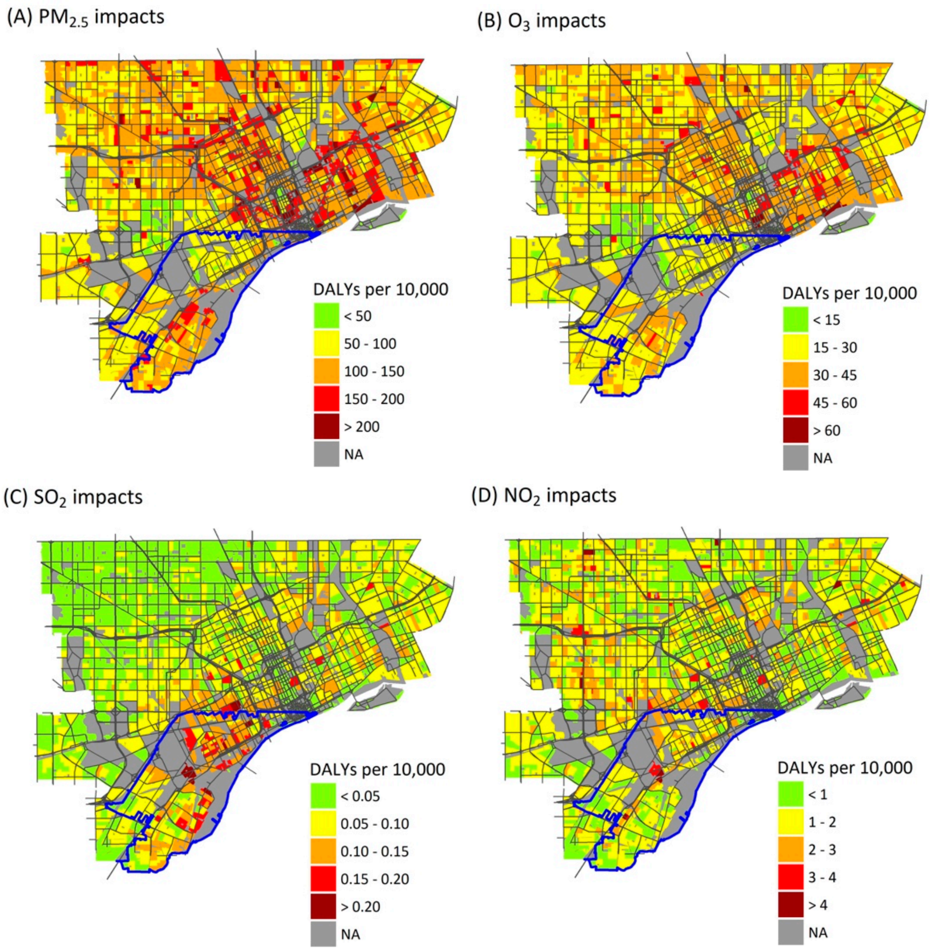

Figure S6. Maps showing the burden of disease (as DALYs per 10,000 per year) attributable to total exposures of (**A**) $PM_{2.5}$, (**B**) ozone, (**C**) SO_2, and (**D**) NO_2. The sub−region of the study area that is in non−attainment of the SO_2 National Ambient Air Quality Standard is shown (blue polygon).

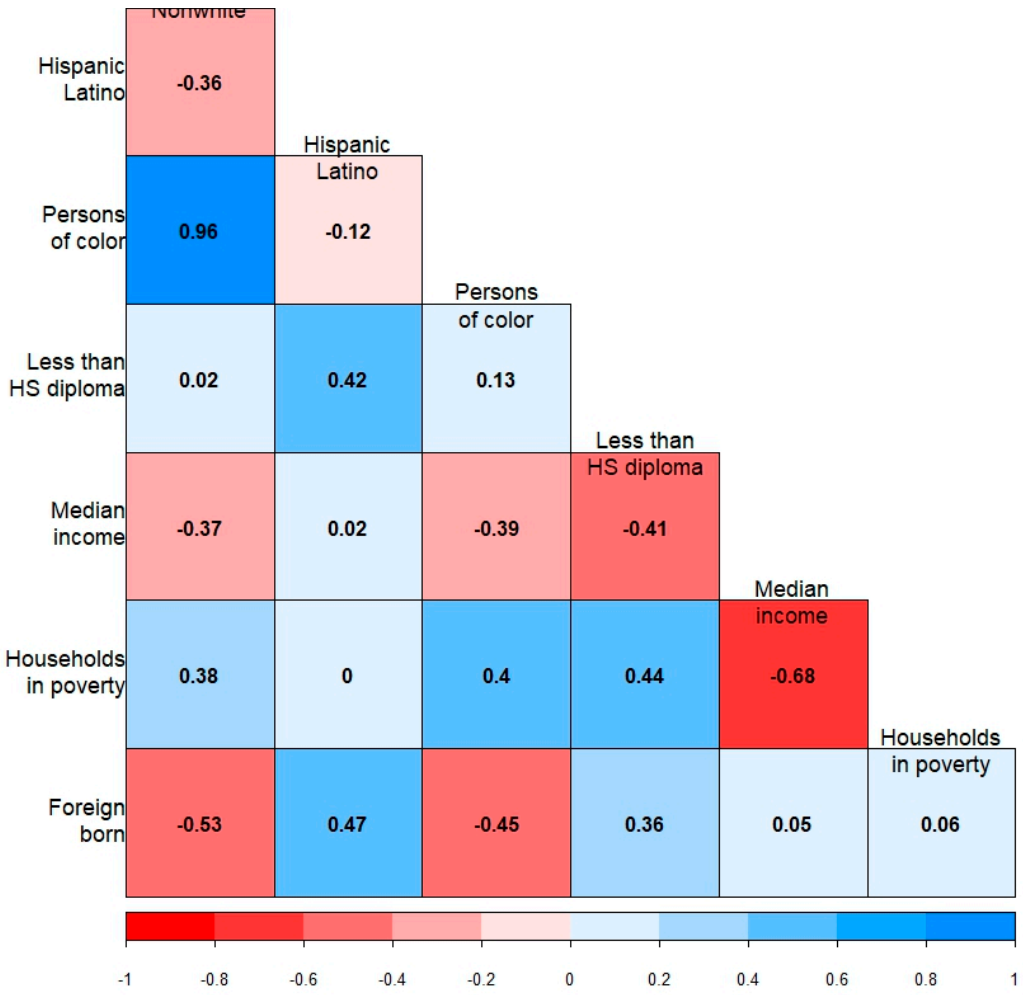

Figure S7. Correlations between block−level demographic and socioeconomic variables in the study area

References

1. Smith, R.L.; Xu, B.; Switzer, P. Reassessing the relationship between ozone and short-term mortality in U.S. urban communities. *Inhal. Toxicol.* **2009**, *21*, 37–61, doi:10.1080/08958370903161612.
2. Mar, T.F.; Koenig, J.Q. Relationship between visits to emergency departments for asthma and ozone exposure in greater Seattle, Washington. *Ann. Allergy Asthma Immunol.* **2009**, *103*, 474–479, doi:10.1016/S1081-1206(10)60263-3.
3. Schildcrout, J.S.; Sheppard, L.; Lumley, T.; Slaughter, J.C.; Koenig, J.Q.; Shapiro, G.G. Ambient Air Pollution and Asthma Exacerbations in Children: An Eight-City Analysis. *Am. J. Epidemiol.* **2006**, *164*, 505–517, doi:10.1093/aje/kwj225.
4. Schwartz, J. Air pollution and hospital admissions for the elderly in Detroit, Michigan. *Am. J. Respir. Crit. Care Med.* **1994**, *150*, 648–655, doi:10.1164/ajrccm.150.3.8087333.
5. Gilliland, F.D.; Berhane, K.; Rappaport, E.B.; Thomas, D.C.; Avol, E.; Gauderman, W.J.; London, S.J.; Margolis, H.G.; McConnell, R.; Islam, K.T.; et. Al. The effects of ambient air pollution on school absenteeism due to respiratory illnesses. *Epidemiology* **2001**, *12*, 43–54.
6. Ostro, B.D.; Rothschild, S. Air pollution and acute respiratory morbidity: An observational study of multiple pollutants. *Environ. Res.* **1989**, *50*, 238–247, doi:10.1016/S0013-9351(89)80004-0.

7. Krewski, D.; Jerrett, M.; Burnett, R.T.; Ma, R.; Hughes, E.; Shi, Y.; Turner, M.C.; Pope III, C.A.; Thurston, G.; Calle, E.E. et. al. Extended follow-up and spatial analysis of the American Cancer Society study linking particulate air pollution and mortality. *Res. Rep. Health Eff. Inst.* **2009**, 5–114.
8. Woodruff, T.J.; Grillo, J.; Schoendorf, K.C. The relationship between selected causes of postneonatal infant mortality and particulate air pollution in the United States. *Environ. Health Perspect.* **1997**, *105*, 608.
9. Sheppard, L. Ambient air pollution and nonelderly asthma hosptial admissions in Seattle, Washington, 1987-1994. *Revis. Anal. Time-Ser. Stud. Air Pollut. Health* **2003**, 227–230.
10. Ito, K. Associations of particulate matter components with daily mortality and morbidity in Detroit, Michigan. *Revis. Anal. Time-Ser. Stud. Air Pollut. Health* **2003**, 143–157.
11. Moolgavkar, S.H. Air pollution and daily deaths and hospital admissions in Los Angeles and Cook counties. *Revis. Anal. Time-Ser. Stud. Air Pollut. Health* **2003**, 183–198.
12. Zanobetti, A.; Franklin, M.; Schwartz, J. Fine particulate air pollution and its components in association with cause-specific emergency admissions in 26 US cities. *Epidemiology* **2008**, *19*, S315–S316.
13. Mar, T.F.; Koenig, J.Q.; Primomo, J. Associations between asthma emergency visits and particulate matter sources, including diesel emissions from stationary generators in Tacoma, Washington. *Inhal. Toxicol.* **2010**, *22*, 445–448, doi:10.3109/08958370903575774.
14. Mar, T.F.; Larson, T.V.; Stier, R.A.; Claiborn, C.; Koenig, J.Q. An analysis of the association between respiratory symptoms in subjects with asthma and daily air pollution in Spokane, Washington. *Inhal. Toxicol.* **2004**, *16*, 809–815, doi:10.1080/08958370490506646.
15. Ostro, B.; Lipsett, M.; Mann, J.; Braxton-Owens, H.; White, M. Air pollution and exacerbation of asthma in African-American children in Los Angeles. *Epidemiology* **2001**, *12*, 200–208.
16. Ostro, B.D. Air pollution and morbidity revisited: A specification test. *J. Environ. Econ. Manag.* **1987**, *14*, 87–98, doi:10.1016/0095-0696(87)90008-8.
17. Yang, Q.; Chen, Y.; Krewski, D.; Burnett, R.T.; Shi, Y.; McGrail, K.M. Effect of short-term exposure to low levels of gaseous pollutants on chronic obstructive pulmonary disease hospitalizations. *Environ. Res.* **2005**, *99*, 99–105, doi:10.1016/j.envres.2004.09.014.
18. Ito, K.; Thurston, G.D.; Silverman, R.A. Characterization of PM2.5, gaseous pollutants, and meteorological interactions in the context of time-series health effects models. *J. Expo. Sci. Environ. Epidemiol.* **2007**, *17*, S45-S60, doi:10.1038/sj.jes.7500627.
19. Li, S.; Batterman, S.; Wasilevich, E.; Wahl, R.; Wirth, J.; Su, F.-C.; Mukherjee, B. Association of daily asthma emergency department visits and hospital admissions with ambient air pollutants among the pediatric Medicaid population in Detroit: Time-series and time-stratified case-crossover analyses with threshold effects. *Environ. Res.* **2011**, *111*, 1137–1147, doi:10.1016/j.envres.2011.06.002.
20. Batterman, S.A.; Lewis, T.; Robins, T.; Mentz, G.; Milando, C.W.; Mukherjee, B. Effects of SO_2 exposures below the national ambient air quality standards in a cohor of children with asthma in Detroit, Michigan. (Manuscript in preparation).
21. Linn, W.S.; Szlachcic, Y.; Gong, H.; Kinney, P.L.; Berhane, K.T. Air pollution and daily hospital admissions in metropolitan Los Angeles. *Environ. Health Perspect.* **2000**, *108*, 427–434.
22. Abridged Life Table, Total Michigan Residents, 2013. Available online: http://www.mdch.state.mi.us/pha/osr/deaths/lifeall.asp (accessed on 15 March 2015).
23. US Environmental Protection Agency, *Regulatory Impact Analysis for the Final Revisions to the National Ambient Air Quality Standards for Particulate Matter*; Office of Air Quality Planning and Standards: Research Triangle Park, NC, USA, 2012.

24. De Hollander, A.E.; Melse, J.M.; Lebret, E.; Kramers, P.G. An aggregate public health indicator to represent the impact of multiple environmental exposures. *Epidemiol. Camb. Mass* **1999**, *10*, 606–617.
25. Centers for Disease Control and Prevention. National Hospital Discharge Survey 2010. Selected Data Tables. Available online: http://www.cdc.gov/nchs/nhds/nhds_tables.htm#number (accessed on 2 December 2014).
26. Murray, C.J. Quantifying the burden of disease: The technical basis for disability-adjusted life years. *Bull. World Health Organ.* **1994**, *72*, 429–445.
27. US Environmental Protection Agency. *Regulatory Impact Analysis of the Final Revisions to the National Ambient Air Quality Standards for Ground-Level Ozone*; US Environmental Protection Agency: Washington, D.C., USA, 2016.
